# Plasma Metabolome Profiling for the Diagnosis of Catecholamine Producing Tumors

**DOI:** 10.3389/fendo.2021.722656

**Published:** 2021-09-07

**Authors:** Juliane März, Max Kurlbaum, Oisin Roche-Lancaster, Timo Deutschbein, Mirko Peitzsch, Cornelia Prehn, Dirk Weismann, Mercedes Robledo, Jerzy Adamski, Martin Fassnacht, Meik Kunz, Matthias Kroiss

**Affiliations:** ^1^Department of Internal Medicine I, Division of Endocrinology and Diabetes, University Hospital, University of Würzburg, Würzburg, Germany; ^2^Core Unit Clinical Mass Spectrometry, University Hospital, Würzburg, Germany; ^3^Chair of Medical Informatics, Friedrich-Alexander University (FAU) of Erlangen-Nürnberg, Erlangen, Germany; ^4^Department of Pediatrics and Adolescent Medicine, University Hospital Erlangen, Erlangen, Germany; ^5^Comprehensive Cancer Center Erlangen-Europäische Metropolregion Nürnberg (CCC ER-EMN), Erlangen, Germany; ^6^Medicover Oldenburg Medizinisches Versorgungszentrum (MVZ), Oldenburg, Germany; ^7^Institute of Clinical Chemistry and Laboratory Medicine, University Hospital Carl Gustav Carus at Technische Universität (TU) Dresden, Dresden, Germany; ^8^Metabolomics and Proteomics Core, Helmholtz Zentrum München, German Research Center for Environmental Health, Neuherberg, Germany; ^9^Hereditary Endocrine Cancer Group, Spanish National Cancer Research Center, Madrid, Spain; ^10^Hereditary Endocrine Cancer Group, Spanish National Cancer Research Center and Centro de Investigación Biomédica en Red de Enfermedades Raras (CIBERER), Madrid, Spain; ^11^Institute of Experimental Genetics, Helmholtz Zentrum München, German Research Center for Environmental Health, Neuherberg, Germany; ^12^Department of Biochemistry, Yong Loo Lin School of Medicine, National University of Singapore, Singapore, Singapore; ^13^Institute of Biochemistry, Faculty of Medicine, University of Ljubljana, Ljubljana, Slovenia; ^14^Cancer Center Mainfranken, University of Würzburg, Würzburg, Germany; ^15^Fraunhofer Institute of Toxicology and Experimental Medicine, Hannover, Germany; ^16^Department of Internal Medicine IV, University Hospital Munich, Ludwig-Maximilians-Universität München, Munich, Germany

**Keywords:** adrenal, pheochromocytoma, paraganglioma, targeted metabolomics, mass spectronomy, catecholamines, machine learning, feature selection

## Abstract

**Context:**

Pheochromocytomas and paragangliomas (PPGL) cause catecholamine excess leading to a characteristic clinical phenotype. Intra-individual changes at metabolome level have been described after surgical PPGL removal. The value of metabolomics for the diagnosis of PPGL has not been studied yet.

**Objective:**

Evaluation of quantitative metabolomics as a diagnostic tool for PPGL.

**Design:**

Targeted metabolomics by liquid chromatography-tandem mass spectrometry of plasma specimens and statistical modeling using ML-based feature selection approaches in a clinically well characterized cohort study.

**Patients:**

Prospectively enrolled patients (n=36, 17 female) from the Prospective Monoamine-producing Tumor Study (PMT) with hormonally active PPGL and 36 matched controls in whom PPGL was rigorously excluded.

**Results:**

Among 188 measured metabolites, only without considering false discovery rate, 4 exhibited statistically significant differences between patients with PPGL and controls (histidine p=0.004, threonine p=0.008, lyso PC a C28:0 p=0.044, sum of hexoses p=0.018). Weak, but significant correlations for histidine, threonine and lyso PC a C28:0 with total urine catecholamine levels were identified. Only the sum of hexoses (reflecting glucose) showed significant correlations with plasma metanephrines.

By using ML-based feature selection approaches, we identified diagnostic signatures which all exhibited low accuracy and sensitivity. The best predictive value (sensitivity 87.5%, accuracy 67.3%) was obtained by using Gradient Boosting Machine Modelling.

**Conclusions:**

The diabetogenic effect of catecholamine excess dominates the plasma metabolome in PPGL patients. While curative surgery for PPGL led to normalization of catecholamine-induced alterations of metabolomics in individual patients, plasma metabolomics are not useful for diagnostic purposes, most likely due to inter-individual variability.

## Introduction

Pheochromocytomas and paragangliomas (PPGL) are defined as catecholamine-producing tumors that arise from chromaffin cells (1). Pheochromocytomas represent more than 80% of all PPGL and are located in the adrenal medulla whereas paraganglioma arise from paravertebral sympathetic ganglia and are most frequently located in the abdomen, chest, and pelvis (2). Paragangliomas deriving from parasympathetic tissue in the head and neck rarely produce hormones (1–3). Predisposing germline mutations, extra-adrenal location, and dopaminergic phenotype are the most relevant risk factors for malignancy (4, 5). Current data suggest that germline mutations are present in up to 40% of all patients with PPGL, with 18 susceptibility genes identified so far. Mutations are most frequently found in genes encoding subunits of succinate dehydrogenase (SDH), von Hippel-Lindau gene (VHL) and rearranged during transfection (RET) gene. As the presence of a germ line mutation was found to be an important factor of prognosis of affected patients, testing is recommended (5–8).

Catecholamine excess leads to a variety of well-known but unspecific symptoms such as hypertension, palpitation, headache and pallor (1, 9) and causes cardio- and cerebrovascular complications. The measurement of plasma free metanephrine (MN), normetanephrine (NMN) and methoxytyramine (MTY) is now a cornerstone of diagnosis and follow-up in clinical practice, providing high diagnostic accuracy when adequate pre-analytics, analytics and reference ranges are applied (2, 10, 11). In recent years mass spectrometry has become the gold standard due to its high analytic sensitivity and specificity (12) not limited on quantification of established markers but showing additionally its usefulness to identify tissue metabolomic profiles *via* MALDI-MSI (13). Nevertheless, the diagnosis of PPGL remains challenging and is often delayed due to lack of consideration of PPGL (4). In addition there is a high risk of false positive test results when strict pre-analytical conditions are not followed.

Metabolomics is the screening for characteristic substances in body fluids and tissue, which serve as direct marker of biochemical activity because they are not exposed to epigenetic regulation and post-translational modifications like proteins or genes and therefore reflect the individual phenotype (14). Untargeted metabolomics allows the identification of numerous molecules without prior knowledge of their presence in predefined groups but has the disadvantage of generating mostly qualitative information on target molecules. On the other hand, quantitation of previously specified molecules is possible by targeted metabolomics. Still, the number of metabolites is typically limited to substances that are precisely characterized by their chemical structure and molecular mass.

In a targeted metabolomics approach, we recently identified significant intra-individual metabolic alterations in patients with PPGL before *vs.* after tumor removal and demonstrated that several of those are related to cardiovascular risk (15). Characterization of the metabolic profile in patients with PPGL might help to understand the metabolic effects of excessive catecholamine levels and harbor additional diagnostic potential.

The aim of our study was to characterize differences in plasma metabolic profile between patients with PPGL and controls with consideration of the secretory phenotype. We applied tandem mass spectrometry using a targeted metabolomics approach and logistic regression modeling to identify discriminative pattern potentially useful for diagnostic workup of PPGL.

## Subjects and Methods

### Subjects

Patients with suspected PPGL were recruited from a single center participating in the Prospective Monoamine-Producing tumor (PMT) study, which has been described in detail previously (2). The diagnosis of PPGL was based on biochemical assessment, imaging, and histology. Follow-up ruled out the presence of PPGLs in patients who served as controls. The latter were matched for sex and age at the date of sampling according to patient data. The study protocol was approved by the Ethics Committee of the University Hospital Würzburg (104/11). All patients provided written informed consent.

### Sample Collection

Plasma samples were collected as described elsewhere (2). Briefly, blood was drawn in the morning after an overnight fast for at least 8h and in a supine position for at least 30 minutes. Patients were instructed to refrain from alcohol, nicotine, decaffeinated and caffeinated beverages for 12 hours as well as avoid acetaminophen five days before sample collection (16). Blood was collected into EDTA or heparinized tubes and placed on ice before centrifugation at 20°C for five minutes at 4000 rpm. Plasma was aliquoted and the samples were stored at -80°C until assayed. Urine collection was performed according to the PMT protocol.

### Mass Spectrometry

Plasma free metanephrines and urine catecholamines were measured as previously described (17–19).

Targeted metabolomics was performed by using the AbsoluteIDQ™-p180 Kit (Biocrates Life Sciences AG, Innsbruck, Austria). The method has been described in detail previously (15, 20, 21) and complies with EMA “Guideline on bioanalytical method validation” (July 21st 2011).The measurement consists of a ultra-high performance liquid chromatography (UHPLC) separation step and a flow injection analysis (FIA) step, both followed by mass spectrometry analyses (LC-MS/MS and FIA-MS/MS). This method enables for measurement of a total of 188 metabolites, of which 42 are included in the LC-MS/MS part (21 amino acids, 21 biogenic amines) and 146 metabolites in the FIA-MS/MS protocol (40 acylcarnitines including free carnitine, 38 phosphatidylcholines with acyl/acyl side chains [PCaa], 38 phosphatidylcholines with acyl/alkyl side chains [PCae], 14 lysophosphatidylcholines [lysoPC], 15 sphingolipids [SM] and the sum of hexoses [H1]).

A volume of 10 µl plasma was used and prepared according to the manufacturer’s manual. Internal standards served as reference for quantification, human reference plasma was included into each batch to ensure quality control, comparability between batch measurements, and normalization of the data (20). Metabolite concentrations are given in µmol/l. LC-MS/MS and FIA-MS/MS were performed by using SCIEX QTRAP^®^ 4500MD MS-system (SCIEX, Darmstadt, Germany) coupled to an Agilent 1290 Infinity UHPLC-system (Agilent, Santa Clara, USA). Analyst^®^ software version 1.6.3 MD (SCIEX, Darmstadt, Germany) was used for data procession. Data was validated and processed with MetIDQ™ software version 5.5.4-DB100 Boron-2623 (Biocrates Life Sciences AG, Innsbruck, Austria).

### Genetics

Genetic data were retrieved from patient records or provided by the CNIO institute in Madrid as a part of the PMT study. Targeted next generation sequencing assay, Sanger sequencing and multiplex ligation-dependent probe amplification or custom array comparative genomic hybridization for deletion detection (22, 23) were applied as appropriate.

### Statistical Analysis

Baseline data are shown as frequencies for categorical variables and as medians with interquartile range (IQR) for numerical variables. Malignancy was defined as the presence of metastases in non-chromaffin organs. The secretory phenotype of PPGL was characterized as noradrenergic and adrenergic according to an established algorithm which has been descripted in detail elsewhere (24). Metabolites with more than 40% of concentrations below the lower limit of quantification (LLOQ) and samples with more than 40% of analytes lower than the lower limit of detection (LOD) were excluded from further analysis. In the remaining metabolites, the non-valid values measured below LLOQ and LOD were left unchanged and included in further analyses. Values with no detectable signal were replaced by (LOD/ √2) x (random number between 0.75-1.25) (25). To detect metabolic alterations associated with catecholamine excess, metabolite values in PPGL and controls were compared. Subgroups were analyzed after stratification for sex (males *vs*. females), BMI (≤ 25 kg/m^2^ *vs* > 25 kg/m^2^) and secretory phenotype (adrenergic *vs*. noradrenergic). Comparisons between groups were analyzed using the Mann-Whitney-U test, significance was defined as p-value <0.05. The calculation of false discovery rate (FDR) corrected p-values was performed according to the method of Benjamini and Hochberg (26). Spearman test was used for correlations between metabolite and catecholamine concentrations. Statistical analyses were performed by SPSS version 25 (IMP, New York, USA) and Prism 7.05 (GraphPad, San Diego, CA, USA), for principal component analysis, MetaboAnalyst (4.0) was used.

### Logistic Regression Modelling

Feature selection models were developed by applying the machine learning methods Elastic net (ELA), Support Vector Machine (SVM) and Gradient Boosting Machine (GBM) using the caret package version 6.0.84. The analysis was done in R (version 3.5.3; script is given online). The data set was normalized using the PreProcess function of the caret package (version 6.0.84). We split the dataset in a training (80%) and validation/test (20%) dataset. We tested each model using repeated 10-fold cross-validation. The variables were selected using the impact that they had on the predictive power of the different models. The models were compared using the predictive values accuracy (correct classification) and kappa (inter-rater reliability; classification including random chance normalization). Identified variables were further analysed using a Wilcoxon test to determine if there is a systematic difference between the conditions (class: PPGL *vs*. control).

## Results

### Patients Characteristics

**Figure 1:**
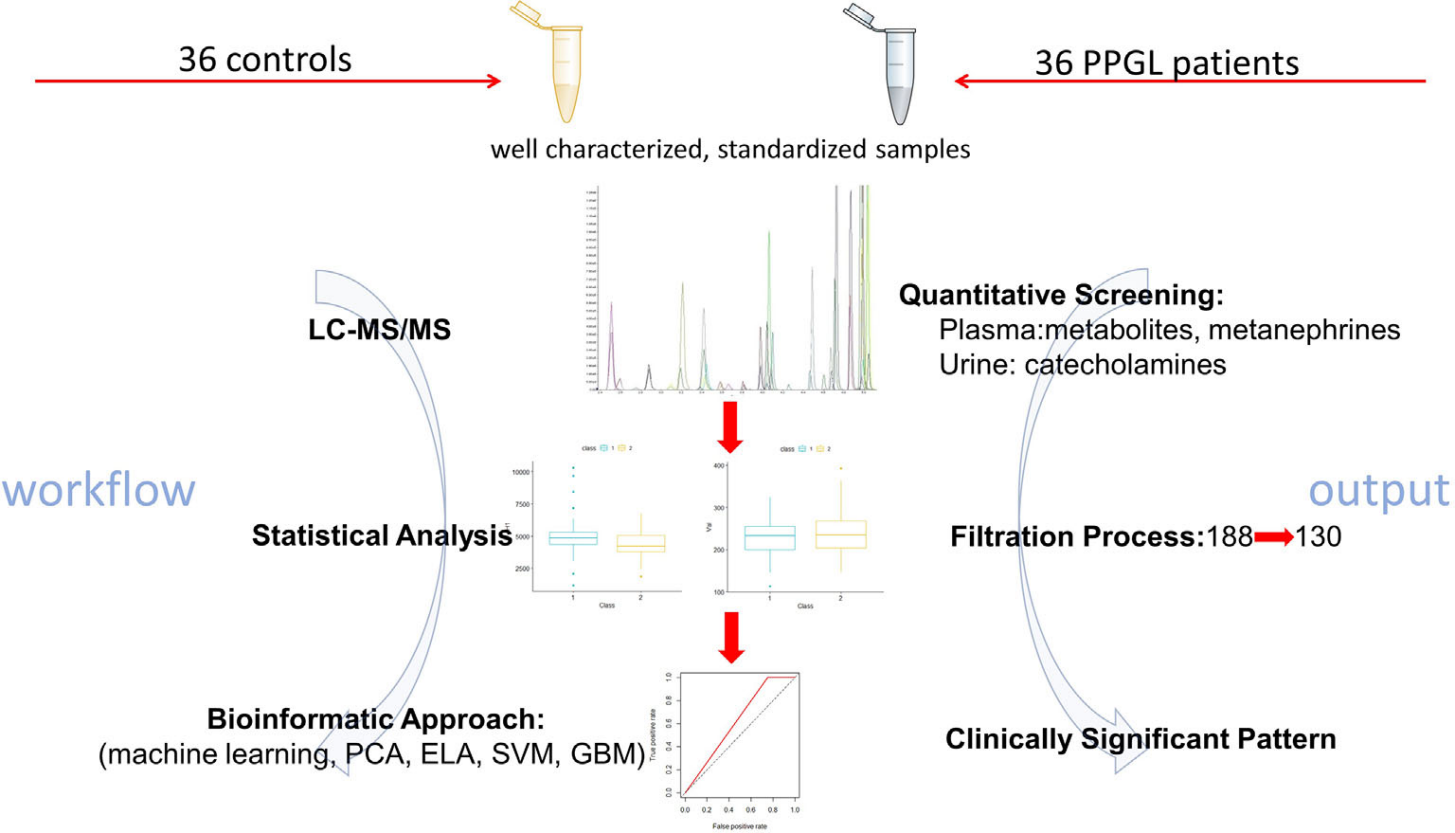
Summary of the workflow leading to identification of significant metabolites *via* liquid chromatography tandem mass spectrometry (LC-MS/MS), in pheochromocytoma and paraganglioma (PPGL),The bioinformatic approach included Elastic net (ELA), Gradient Boosting Machine (GBM), Support Vector Machine (SVM) and Principal component analysis (PCA) approaches.

The study workflow is depicted in **Figure 1**. 36 patients with confirmed PPGL prior any specific treatment and controls matched for sex and age at date of sample were selected (**Table 1**). In controls, PPGL was suspected based on the incidental finding of an adrenal mass upon imaging for an unrelated condition (n=21), signs and symptoms suggestive for PPGL (n=9) or therapy resistant hypertension (n=6) but excluded by normal follow-up biochemistry, negative imaging, resolved signs and symptoms or an alternative diagnosis (17). Other endocrinological causes for resistant hypertension or adrenal incidentalomas such as hyperaldosteronism, acromegaly, hyperthyroidism, hyperparathyroidism, or Cushing’s syndrome were excluded in all subjects.

**Table 1:** Patient characteristics stratified by patients and controls.

	PPGL	Controls	P value
Subjects, n	36	36	
Females, n (%)	17 (47)	17 (47)	
Extra-adrenal tumor location, n (%)	7 (19)		
Malignant tumor, n (%)	11 (31)		
Tumor size, d [cm]	4.1 (3.3-6.1)		
BMI, [kg/m^2^]	25.2 (23.6-26.7)	28.6 (26.0-31.2)	0.043
AHT, n (%)	14 (39)	24 (67)	
Diabetes mellitus, n (%)	6 (17)		
Adrenergic phenotype, n (%)	15 (42)		
PHEO, n	15		
PGL, n	0		
Follow-up			
6 months, n (%)		18 (50)	
24 months, n (%)		11 (31)	
Plasma data (n=72)			
Time between sampling and metabolomics measurement (days)	1164 (922-1407)	1211 (976-1447)	0.971
Age at date of sample	50.7 (41.7-61.4)	50.9 (43.7-62.2)	0.884
MN [pg/ml]	66.7 (31.0-596.2)	28.4 (21.0-45.4)	<0.001
NMN [pg/ml]	1144.4 (561.4-2327.8)	82.8 (62.3-121.7)	<0.001
MTY [pg/ml]	14.1 (7.8-111.6)	5.5 (3.4-8.8)	<0.001
Urine data (n=58)]			
Age at date of sample	53.7 (43.3-61.7)	52.5 (46.8-62.9)	0.953
Free NE [µg/day]	75.0 (38.0-160.9)	20.6 (15.0-37.9)	<0.001
Free EPI [µg/day]	9.6 (2.8-34.6)	4.0 (2.3-5.9)	0.011
Free DA [µg/day]	217.1 (144.3-288.0)	218.5 (165.1-249.8)	0.767
Genetic screening (germline) [N=33]			
Unknown	3		
Wild type	24		
SDHB	2		
NF1	3		
VHL	1		
Antihypertensive medication, n (%)			
Alpha-blocker	14 (39)	10 (27)	
Beta-blocker	16 (44)	12 (33)	
Diuretics	5 (14)	5 (14)	
ACE-inhibitor/AT1-antagonist	10 (27)	8 (22)	
Calcium channel blocker	5 (14)	10 (27)	

Numerical variables data are represented as median with range (inter-quartile) in brackets. For categorical variables, absolute and percentage values are given.

AHT, arterial hypertension; BMI, body mass index; DA, dopamine; EPI, epinephrine; MN, metanephrine; MTY, 3-methoxytyramine; NE, norepinephrine; NMN, normetanephrine; PPGL, pheochromocytoma/paraganglioma.

Complete urinary catecholamine data (31/36 controls, 27/36 PPGL) and genetic data (33/36 PPGL) were available in a subset of individuals. There were no statistically significant differences between groups concerning age and time between sampling and measurement of metabolomics while a statistically significant difference in Body mass index (in kg/m^2^) was present in PPGL (25.2 [23.6–26.7]) *vs* controls (28.6 [26.0–31.2], p=0.043). Plasma markers of catecholamine excess were significantly increased in PPGL.

### Targeted Metabolomics PPGL *vs.* Controls

**Figure 2:**
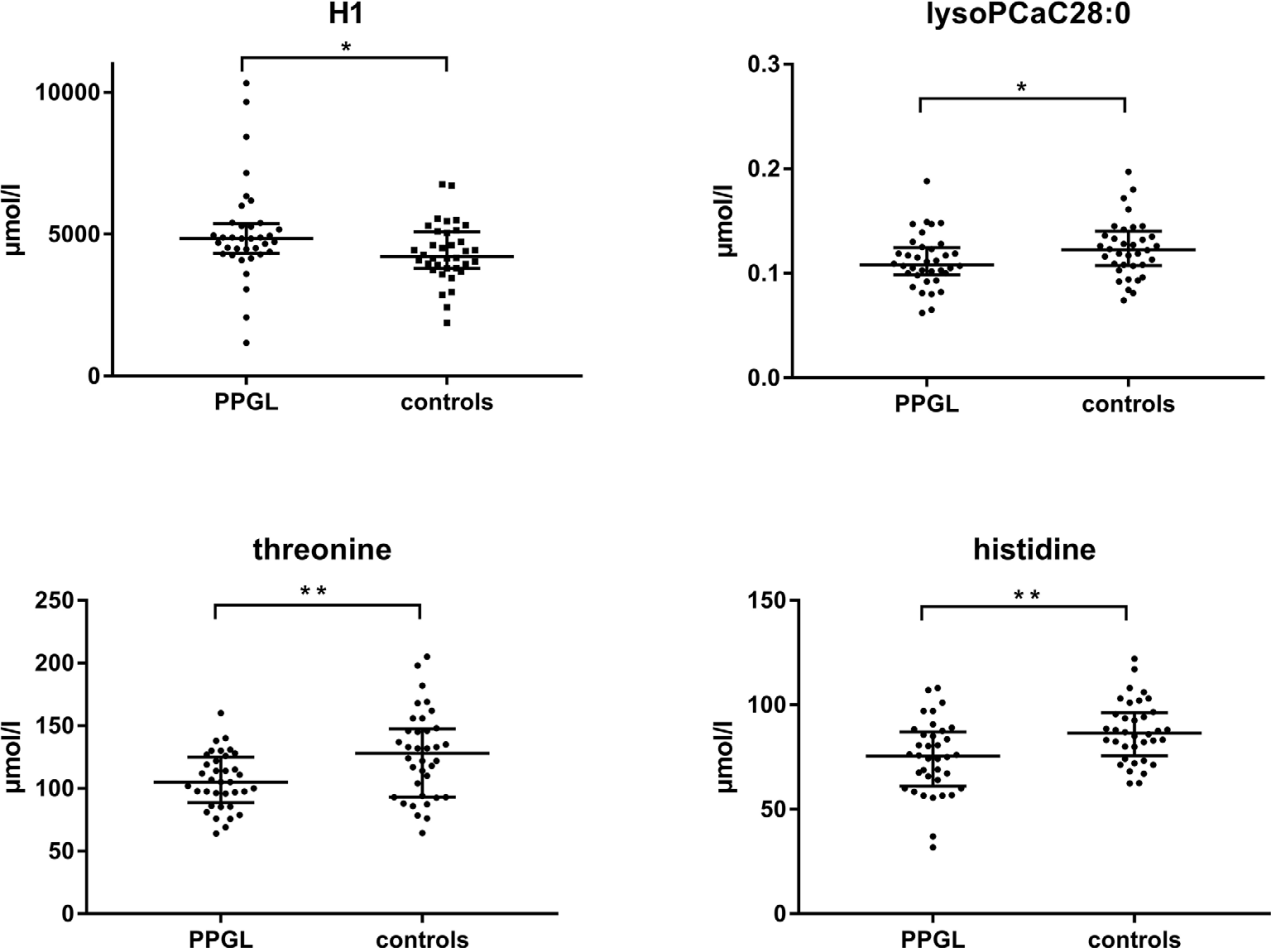
Scatter plot of median plasma levels from metabolites measured by LC-MS/MS with significant differences between PPGL patients and controls. (Mann-Whitney-U-test, p < 0.05). H1, sum of hexoses; lysoPC, lysophosphatidylcholine; PPGL, pheochromocytoma and paraganglioma. *<0.05, **<0.01.

Overall, 130 of 188 measured metabolites were included in the statistical analysis (**Supplemental Data**). However, only when p-values were not corrected for FDR, four of them showed significantly different concentrations between the two groups. In PPGL *vs*. controls (**Figure 2**) the amino acids histidine (75.40 [61.03-87.05] *vs.* 86.40 [75.63-96.35] µmol/l, p=0.004) and threonine (105.00 [88.57-125.00] *vs.* 128.00 [93.32-147.50] µmol/l, p=0.008) were significantly lower, while lyso PC a C28:0 (0.11 [0.10-0.12] *vs*. 0.12 [0.11-0.14] µmol/l, p=0.044) was only slightly decreased. On the opposite, the sum of hexoses was significantly higher in PPGL patients compared to controls (4844.00 [4325.50-5364.50] *vs*. 4215.50 [3791.00-5086.00] µmol/l, p=0.018). The plasma concentrations of biogenic amines, acylcarnitines, and sphingolipids were comparable between PPGL and controls.

### Correlation With Plasma Metanephrine and Urine Catecholamine Values

**Figure 3:**
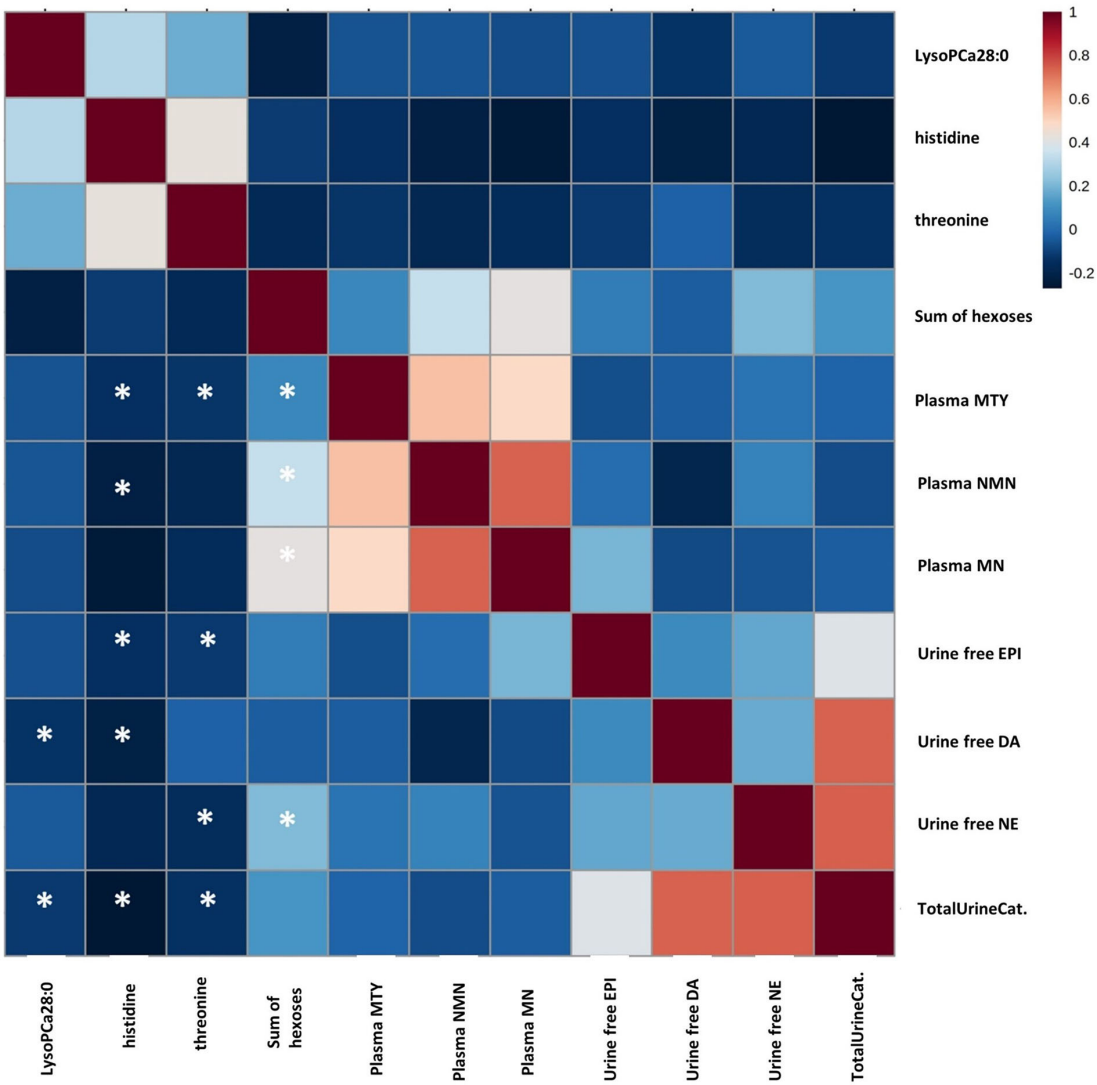
Correlation between plasma concentrations of metanephrine, normetanephrine, and methoxytyramine, 24h urine concentrations of catecholamines, and plasma concentrations of significantly altered metabolites in PPGL patients. Spearmen-coefficient r_s_ is presented by color coding (positive correlation: red; negative correlation: blue). An Asterisk indicates a statistically significant correlation at the level of (p < 0.05). DA, dopamine; EPI, epinephrine; MN, metanephrine; MTY, methoxytyramine; NE, norepinephrine; TotalUrineCat, Total urine catecholamines.

The association of metabolic changes with catecholamine excretion has already been demonstrated (15). Therefore, we correlated altered metabolites with urinary catecholamines, which represent the biologically active form, and MN, NMN and MTY in plasma as non-functional disease markers (**Table 2** and **Figure 3**). Histidine showed a significant negative correlation with plasma NMN and plasma MTY, as well as with urine free epinephrine (EPI) and urine free dopamine (DA). Threonine was negatively correlated with plasma MTY, urine free norepinephrine (NE), and urine free EPI. LysoPC a C28:0 was negatively associated with urine free DA, whereas the sum of hexoses showed a positive correlation with all plasma metanephrines and with urine free NE. Furthermore, Histidine, threonine and lysoPC C:28 revealed a negative correlation with the total urinary catecholamines.

**Table 2:** Plasma levels of significant altered metabolites (p ≤ 0.05) in patients with PPGL in comparison to controls including subgroup analysis and the correlation with free plasma metanephrines and 24h urinary free catecholamine excretion values.

	PPGL	Controls	P value	Correlations (Spearman r_s_)						
				Plasma			Urine			
				NMN	MN	MTY	NE	EPI	DA	total catecholamine
**All patients**										
Histidine	75.40 (61.03-87.05)	86.40 (75.63-96.35)	0.004	**-0.287**	-0.219	**-0.242**	-0.239	**-0.408**	**-0.300**	**-0.407**
Threonine	105.00 (88.57-125.00)	128.00 (93.32-147.50)	0.008	-0.229	-0.054	**-0.266**	**-0.255**	**-0.304**	-0.161	**-0.275**
lysoPC a C28:0	0.11 (0.10-0.12)	0.12 (0.11-0.14)	0.044	-0.169	-0.147	-0.144	-0.212	-0.219	**-0.260**	**-0.269**
Hexose	4844.00 (4325.50- 5364.50)	4215.50 (3791.00-5086.00)	0.018	**0.337**	**0.276**	**0.339**	**0.437**	0.145	-0.046	0.221
										
**Males**										
Threonine	102.00 (85.50-127.00)	132.00 (104.00-156.00)	0.008	**-0.328**	-0.206	-0.226	-0.269	**-0.345**	-0.085	-0.212
lysoPC a C16:1	1.68 (1.09-1.79)	1.89 (1.54-2.16)	0.025	-0.295	0.163	-0.147	-0.329	0.042	-0.102	-0.184
PC ae C30:2	0.07 (0.06-0.08)	0.06 (0.06-0.07)	0.040	0.244	-0.168	0.071	0.185	-0.181	-0.208	-0.040
SM (OH) C14:1	4.77 (3.20-5.43)	3.46 (2.59-4.38)	0.040	0.266	-0.157	-0.060	0.071	-0.151	-0.253	-0.084
Hexose	4845.00 (4489.00- 5285.00)	4079.00 (3789.00-5131.00)	0.050	**0.381**	0,068	0.306	**0.425**	-0.020	-0.097	0.206
										
**Females**										
Histidine	64.10 (56.70-78.30)	85.90 (71.70-104.50)	0.001	**-0.428**	-0.339	-0.301	-0.364	**-0.434**	0.294	**-0.479**
lysoPC a C20:4	3.95 (3.50-4.95)	2.62 (2.44-3.54)	0.006	**0.471**	0.273	**0.438**	**0.537**	**0.412**	0.302	**0.538**
PC aa C36:4	203.00 (165.00-249.50)	160.00 (124.00-211.00)	0.041	**0.356**	**0.514**	**0.525**	0.117	0.022	-0.256	-0.123
PC aa C38:4	94.10 (77.35-107.00)	82.60 (64.40-91.55)	0.049	0.325	**0.535**	**0.508**	0.120	0.197	-0.093	0.002
PC ae C38:1	0.37 (0.28-1.24)	0.71 (0.47-1.69)	0.022	**-0.368**	**-0.474**	-0.137	-0.353	**-0.531**	-0.048	-0.048
										
**Adrenergic**										
Glycin	221.00 (182.00-373.00)	167.00 (145.00-192.00)	0.007	**0.464**	**0.490**	0.258	0.280	0.376	0.108	0.297
Histidine	69.00 (58.40-76.10)	85.10 (71.30-93.60)	0.004	**-0.597**	**-0.494**	**-0.535**	**-0.412**	**-0.499**	-0.315	**-0.459**
lysoPC a C20:4	3.99 (3.37-5.18)	3.10 (2.46-4.04)	0.019	0.353	0.317	0.082	**0.461**	**0.477**	0.087	0.283
LysoPC a C28:0	0.11 (0.08-0.11)	0.13 (0.11-0.14)	0.026	**-0.371**	-0.236	-0.336	-0.345	-0.327	-0.211	-0.297
										
**Noradrenergic**										
C0	35.60 (28.70-41.15)	43.00 (34.05-48.70)	0.042	-0.226	0.201	-0.234	-0.114	-0.089	-0.342	-0.290
Asparagine	37.20 (32.00-41.95)	42.00 (38.65-48.05)	0.013	-0.252	0.129	-0.124	-0.254	-0.060	-0.116	-0.193
Threonine	112.00 (96.20-127.50)	133.00 (120.00-151.00)	0.002	-**0.344**	0.064	**-0.351**	**-0.472**	-0.199	-0.307	**-0.494**
ADMA	0.51 (0.39-0.80)	0.62 (0.55-0.91)	0.048	-0.258	-0.041	-0.132	**-0.399**	-0.029	-0.001	-0.195

Plasma levels of significant altered metabolites are given in µmol/l. Metabolomics data is expressed as median with range (inter-quartile) in brackets. Mann-Whitney-U test was performed, and p-values (two-tailed) are reported. The r_s_-value represents the Spearman correlation coefficient. Significant correlations are marked bold.

DA, dopamine; EPI, epinephrine; MN, metanephrine; MTY, 3-methoxytyramine; NE, norepinephrine; NMN, normetanephrine; PHEO, pheochromocytoma; PGL, paraganglioma; PPGL, pheochromocytoma/paraganglioma.

### Subgroup Analyses

Despite the small group sizes, we also explored differences in subgroups. However, these were significant only without FDR correction. If only males were taken into account (n=19), patients with PPGL had lower levels of threonine (102.00 [85.50-127.00] *vs*. 132.00 [104.00-156.00] µmol/l, p=0.008) and higher levels of H1 (4845.00 [4489.00-5285.00] *vs*. 4079.00 [3789.00-5131.00] µmol/l, p=0.050) than controls. In addition, alterations in three additional metabolites were present: Lyso PC a C16:1 showed lower levels in PPGL (1.68 [1.09-1.79] *vs*. 1.89 [1.54-2.16] µmol/l, p=0.025), whilst PC ae C30:2 and SM (OH) C14:1 had significantly higher levels compared to controls (0.07 [0.06-0.08] *vs*. 0.06 [0.06-0.07] µmol/l, p=0.040 and 4.77 [3.20-5.43] *vs*. 3.46 [2.59-4.38] µmol/l, p=0.040).

In the subgroup with BMI below or equal 25 kg/m^2^ the PPGL patients (n=22) exhibited lower level of histidine (75.20 [64.35-85.90] *vs*. 87.50 [82.40-103.00] μmol/l, p=0.006) and higher level of H1 (4773.00 [4357.00-5311.50] *vs*. 4146.00 [3789.00-4428.00] µmol/l, p=0.003) than controls (n=15). In the subgroup with BMI above 25 kg/m^2^ PPGL patients (n=12) had higher level of SM OH C22:1 (7.09 [5.49-8.29] *vs*. 5.88 [4.81-6.64] µmol/l, p=0.048) and SM OH C22:2 (5.66 [5.16-6.81] *vs*. 4.90 [3.94-5.51] µmol/l, p=0.036). Octadecenoylcarnitine (0.099 [0.088-0.131] *vs*. 0.135 [0.102-0.203] µmol/l, p=0.048), octadecadienylcarnitine 0.029 [0.023-0.034] *vs*. 0.035 [0.029-0.064] µmol/l, p=0.044) and histidine 71.10 [58.80-85.17] *vs*. 84.20 [71.50-93.80] µmol/l, p=0.036), were lower in PPGL patients than in controls (n=20), The same applies for ornithine (60.75 [54.10-71.30] *vs*. 92.60 [58.10-124.50] µmol/l, p=0.036) and threonine (98.85 [85.50-122.25] vs. 127.00 [93.33-153.50] µmol/l, p=0.036).

Female PPGL patients exhibited higher levels of lysoPC a C20:4 (3.95 [3.50-4.95] *vs*. 2.62 [2.44-3.54] µmol/l, p=0.006), PC aa C36:4 (203.00 [165.00-249.50] *vs*. 160.00 [124.00-211.00] µmol/l, p=0.041) and PC aa C38:4 (94.10 [77.35-107.00] *vs*. 82.60 [64.40-91.55] µmol/l, p=0.049) as well as lower values of PC ae C38:1 (0.37 [0.28-1.24] *vs*. 0.71 [0.47-1.69] µmol/l, p=0.022) and histidine (64.10 [56.70-78.30] *vs*. 85.90 [71.70-104.50] µmol/l, p=0.001).

Analyzing only the subgroup of adrenergic phenotypes, we found increased levels of glycin (221.00 [182.00-373.00] *vs*. 167.00 [145.00-192.00] µmol/l, p=0.007) and lysoPC a C20:4 (4.00 [3.37-5.18] *vs*. 3.10 [2.46-4.04] µmol/l, p=0.019] combined with decreased levels of lysoPC a C28:0 (0.11 [0.08-0.11] *vs*. 0.13 [0.11-0.14] µmol/l, p=0.026) in patients with PPGL compared to controls. Histidine, which had lower levels in the entire group as well as in females, showed also lower concentrations in adrenergic phenotypes (69.00 [58.40-76.10] *vs*. 85.10 [71.30-93.60] µmol/l, p=0.004). In PPGL with noradrenergic phenotype we found lower concentrations of C0 (35.60 [28.70-41.15] *vs*. 43.00 [34.05-48.70] µmol/l, p=0.042), asparagine (37.20 [32.00-41.95] *vs*. 42.00 [38.65-48.05] µmol/l, p=0.013), threonine (112.00 [96.20-127.50] *vs*. 133.00 [120.00-151.00] µmol/l, p=0.002) and ADMA (0.51 [0.39-0.80] *vs*. 0.62 [0.55-0.91] µmol/l, p=0.048).

### Feature Selection Using Machine Learning Techniques and Principal Component Analysis

**Figure 4:**
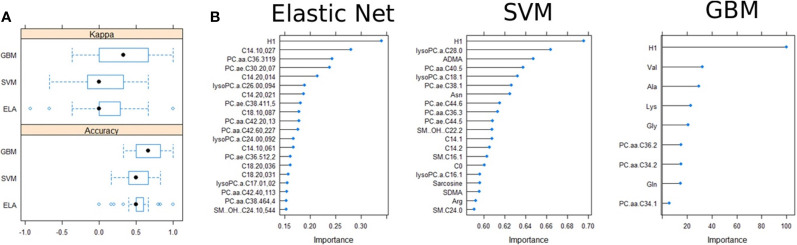
Variable selection in the training dataset. **(A)** Accuracy and Kappa comparisons of the three models used in feature selection. **(B)** Importance of the variable selection and out-of-bag predictive performance estimation.

The machine learning models GBM, ELA, and SVM were run to determine features which are important to class prediction. Each of the models was run with 10-fold cross-validation on the training dataset, the features which contributed most to class prediction were obtained and the models were compared based on their estimated performance. The GBM, ELA and SVM had an estimated accuracy of 0.67, 0.53 and 0.53 respectively (Kappa: GBM 0.33, ELA 0.06 and SVM 0.08; **Figure 4A**). ELA and SVM selected 20 variables, whereas the GBM only selected 9 variables (**Figure 4B**). The GBM had the best estimated predictive value of which hexose showed the largest contribution to diagnosis, calculated using an out-of-bag estimate of the improvement in predictive performance. Comparing the selected variables, only H1 was shared between the 3 modelling algorithms. Furthermore, for GBM each of the selected variables was evaluated for difference (**Supplemental Data**), which found that only the H1 predictor has a significant difference between the two classes. Testing for the class prediction in the validation dataset showed limited predictive value for all models.

Similar analyses were also preformed to select predictors of catecholamine producing tumor types (pheo *vs*. PGL *vs*. control) phenotypes (adrenergic *vs*. noradrenergic *vs*. control) and malignancy (benign *vs*. malignant *vs*. control). The predictors that were selected in all these cases were the same as from the initial analysis, but further investigation of the distribution of the predictors showed a much weaker difference between the groups (diagnosis, phenotype and malignancy) when compared with the distribution between the difference classes (PPGL *vs*. control). GBM selected the same variables in each section (**Supplemental Data**). However, accuracy was always lower than 60%. The most commonly selected variable H1 showed difference between different analyses in phenotype p=0.10 between control and adrenergic/noradrenergic, but p=0.68 between the two states. In malignancy we calculated p=0.11 between benign and control and p=0.08 between benign and malignant, but p=0.76 between control and malignant.

The score plots obtained from principal component analysis models after logarithmic normalization of the entire dataset and subgroup (adrenergic, noradrenergic) demonstrated that the groups were not well discriminated (**Supplemental Data**).

## Discussion

In our study 36 PPGL patients and matched controls were analyzed with targeted metabolomics. Despite the highly standardized sampling conditions and quantitative liquid chromatography-tandem mass spectrometry, we only found metabolites with significant differences between PPGL patients and matched controls when no correction for FDR was performed. After correction for multiple comparisons, the statistical significance was not retained for all metabolites. Classifying substances as significant without FDR-correction was reasonable in our preliminary setting as the focus was to identify potentially relevant metabolites, which have to be validated in further studies. In this approach it may be preferable to explore leads that may turn out to be wrong than loosing promising markers in early state by stringent statistical criteria, as has been argued by others (27). By using machine learning, we failed to establish metabolic signatures associated with PPGL diagnosis, indicating little value of such targeted metabolomics approach for diagnostic purposes in PPGL at variance to plasma metanephrin, normetanephrin and 3-methoxytyramin which have proven excellent sensitivity and specificity when performed with appropriate preanalytics, analytics and reference intervals.

It is well known that catecholamine excess leads to a diabetogenic state (15, 28–30) and we accordingly found higher levels of hexoses in PPGL patients. This reflects increased glycogen catabolism, glucagon release and gluconeogenesis finally leading to the high prevalence of diabetes mellitus (21-37%) in PPGL patients.

Erlic et al. compared the same metabolite spectrum in PPGL patients before and post-surgery and found lower histidine levels in preoperative samples (15). A low histidine level is linked to type 2 diabetes, increased inflammation and cardiovascular disease (31–33), potentially explaining (at least in part) such effects in PPGL patients (31–34).

The negative correlation of histidine with plasma NMN and MTY, urine free EPI and DA in our cohort suggests a catabolic phenotype which has been linked to proinflammatory mediators as well (35). In this regard it is noteworthy that BMI was significantly lower in patients compared to controls. While one may argue that this reflects an imbalance in the matching of base line characteristics, it may rather reflect the catabolic effect of PPGL.

Sex specific differences in metabolic pattern found by Erlic et al. by comparing intra-individual metabolite profiles prior and after surgical tumor removal were confirmed here even if the results should be treated with caution due to the small number of patients in the subgroups (15).

Significant changes between PPGL and controls observed in threonine and histidine are in accordance with other studies that focused on cancer (36, 37). For example, Miyagi et al. also showed decreased levels of histidine in patients with gastric, colorectal, lung and breast cancer, while threonine was lower in gastric as well as colorectal cancer and higher in bronchial carcinoma. The mechanistic background and clinical significance are still subject of discussion (37).

ML approaches imply the need for a substantial amount of data for the development of clinical useful diagnostic models, thus limits the application potential in rare diseases such as PPGL. We overcome this by combining statistical correlation analysis with 3 different feature selection algorithm and applied several validation steps such as data splitting and 10-fold cross-validation on a non-linear and large dimensional variable (130 metabolites) dataset (38). However, even the best performing model (GBM with 9 selected metabolites) showed low predictive values within the test dataset. The GBM model shows a higher sensitivity (87.5%) compared to the other models but was outperformed by ELA and SVM in terms of specificity. Given the low accuracy and no significant p-value, the models show low ability to distinguish between PPGL and control. Similarly, model selection based on diagnosis, phenotype and malignancy predictors showed a much weaker difference between the groups (accuracy always <60%). Given the small size of the disease subsets and the poor significance found in the distributions between each group subset, the confidence with which the features can be used to differentiate between the different group subsets was limited.

It might be argued that a larger study has enabled us to identify a distinct phenotype using ML-based selection approaches which then could possibly include a larger number of features under study. We do not share this point of view because any test applied in PPGL for diagnostic purposes requires a much higher sensitivity and specificity compared to that found in our pilot study to be clinically meaningful. This is particularly true when the generally low pre-test likelihood of a PPGL and the prevalence of diabetes and catabolism in an unselected population is considered. Consequently, a further analysis of the total cohort of the PMT study regarding this pilot study does not seem reasonable.

PPGL-associated mutations in genes involved in the Krebs cycle and electron transport chain have shown to translate into characteristic tumoral metabolic changes (39). Tumors caused by mutations in the SDH genes that belong to this cluster 1 tumors exhibit an increased succinate fumarate ratio in tumor tissue (23, 40, 41) which leads to a pseudo-hypoxic phenotype that downstream activates hypoxia induced factor signaling and angiogenesis.

Recently, Wallace et al. showed that assessment of Krebs cycle related metabolites by using LC-MS/MS in addition to immunohistochemistry improved the diagnosis of SDH impairment at a functional level (42). These tumoral metabolic pathway alterations translate into characteristic secretory pattern of catecholamine metabolites which contribute to the diagnosis of malignancy (39, 43–46). Of note, they also appear to have therapeutic relevance (39, 43–45).

Taken together, we confirmed previous findings of metabolic alterations caused by PPGL related catecholamine excess by comparing PPGL patients and controls. We applied machine learning algorithms, but these failed to provide feature-selection signatures that may be useful for PPGL diagnosis in clinical routine. Still, our study broadens and complements the understanding of changes in the metabolic profile of patients with PPGL.

## Data Availability Statement

The raw data supporting the conclusions of this article will be made available by the authors, without undue reservation.

## Ethics Statement

The studies involving human participants were reviewed and approved by Ethics committee of the Medical Faculty, University of Würzburg. Written informed consent to participate in this study was obtained from all study participants and, in the case of minors, from their legal representatives.

## Author Contributions

JM: Data acquisition, data analysis, data interpretation, and writing of first paper draft. MK: Conception of the work, data acquisition, data analysis, writing of first paper draft, and revising the work critically for important intellectual content. OR-L: Data analysis, data interpretation, writing of first paper draft, and revising the work. TD: Data interpretation and revising the work critically for important intellectual content. MP: Data acquisition, data analysis, data interpretation, and revising the work critically for important intellectual content. CP: Data acquisition and revising the work critically for important intellectual content. DW: Revising the work critically for important intellectual content. MR: Data acquisition and revising the work critically for important intellectual content. JA: Data acquisition and revising the work critically for important intellectual content. MF: Planning and design of the work and revising the work critically for important intellectual content. MKu: Data analysis, data interpretation, and drafting and revising it critically for important intellectual content. MKr: Conception, planning and design of the work, data analysis, data interpretation, drafting and revising it critically for important intellectual content, important intellectual content. MKu and MKr contributed equally to this study. All authors contributed to the article and approved the submitted version.

## Funding

This work was supported by the German Research Council (DFG) project 314061271 (SFB/CRC Transregio 205: The adrenal – central relay in health and disease).

## Conflict of Interest

The authors declare that the research was conducted in the absence of any commercial or financial relationships that could be construed as a potential conflict of interest.

## Publisher’s Note

All claims expressed in this article are solely those of the authors and do not necessarily represent those of their affiliated organizations, or those of the publisher, the editors and the reviewers. Any product that may be evaluated in this article, or claim that may be made by its manufacturer, is not guaranteed or endorsed by the publisher.

## Abbreviations

a, acyl; aa, diacyl; ae, acyl-alkyl; C x:y, indicates the lipid chain composition where “x” is the number of carbons and “y” the number of bonds; C, carbon; DA, dopamine; ELA, Elastic net; EPI, epinephrine; FDR, false discovery rate; FIA, flow injection analysis; GBM, Gradient Boosting Machine; H1, sum of hexoses; IQR, interquartile range; LC-MS/MS, liquid chromatography tandem mass spectrometry; LLOQ, lower limit of quantification; LOD, lower limit of detection; lysoPC, lysophosphatidylcholine; MN, metanephrine; MTY, methoxytyramine; NE, norepinephrine; NMN, normetanephrine; PC, phosphatidylcholine; PMT, Prospective Monoamine-producing Tumor Study; PPGL, pheochromocytoma/paraganglioma; SDH, succinate dehydrogenase; SM, sphingolipids; SVM, Support Vector Machine; UHLPC, ultra-high performance liquid chromatography.
