# Supplementary material for: Plasma Metabolome Profiling for the Diagnosis of Catecholamine Producing Tumors
Source: Front Endocrinol (Lausanne). 2021 Sep 7;12:722656. doi: 10.3389/fendo.2021.722656 (PMC8453166; doi:10.3389/fendo.2021.722656)

**Supplemental data**

**secretory type**

PCA analysis (including 95% confidence regions): (1=adrenergic, 2=noradrenergic, 3= control) of 130 metabolites (log normalization)


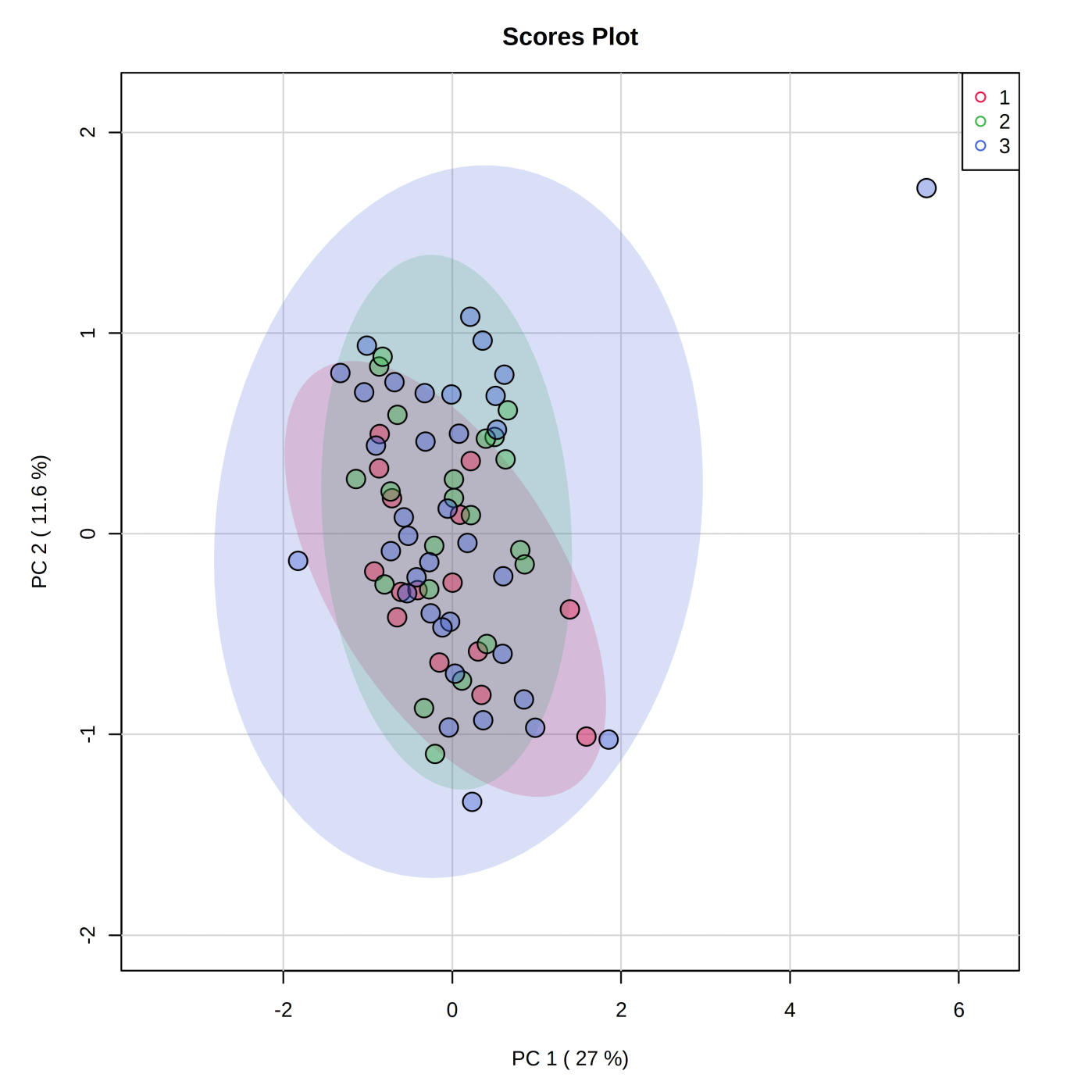


corresponding Biplot (arrows showing importance of parameters)


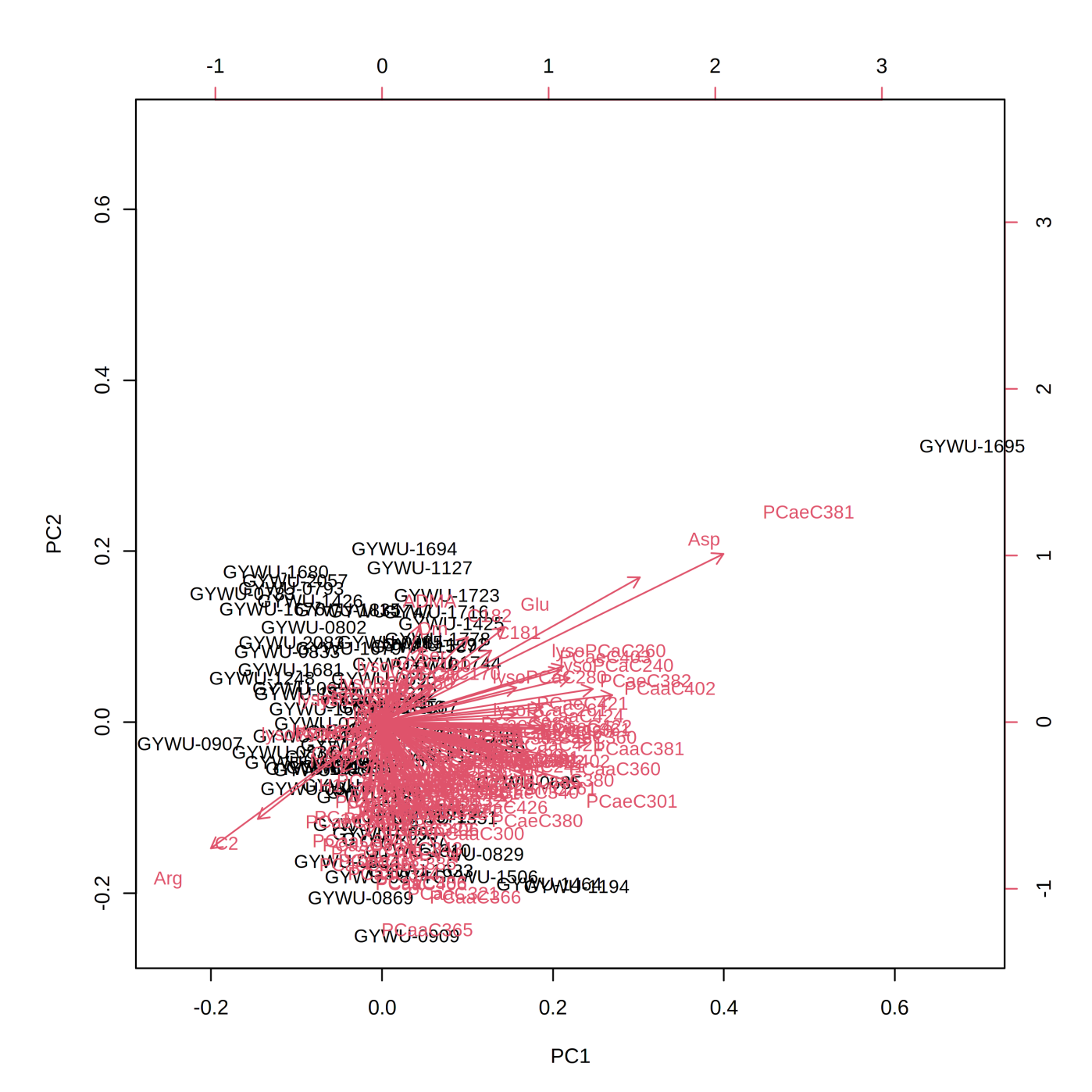


**malignancy**

PCA analysis (including 95% confidence regions): (1=benign, 2=malignant, 3= control) of 130 metabolites (log normalization)


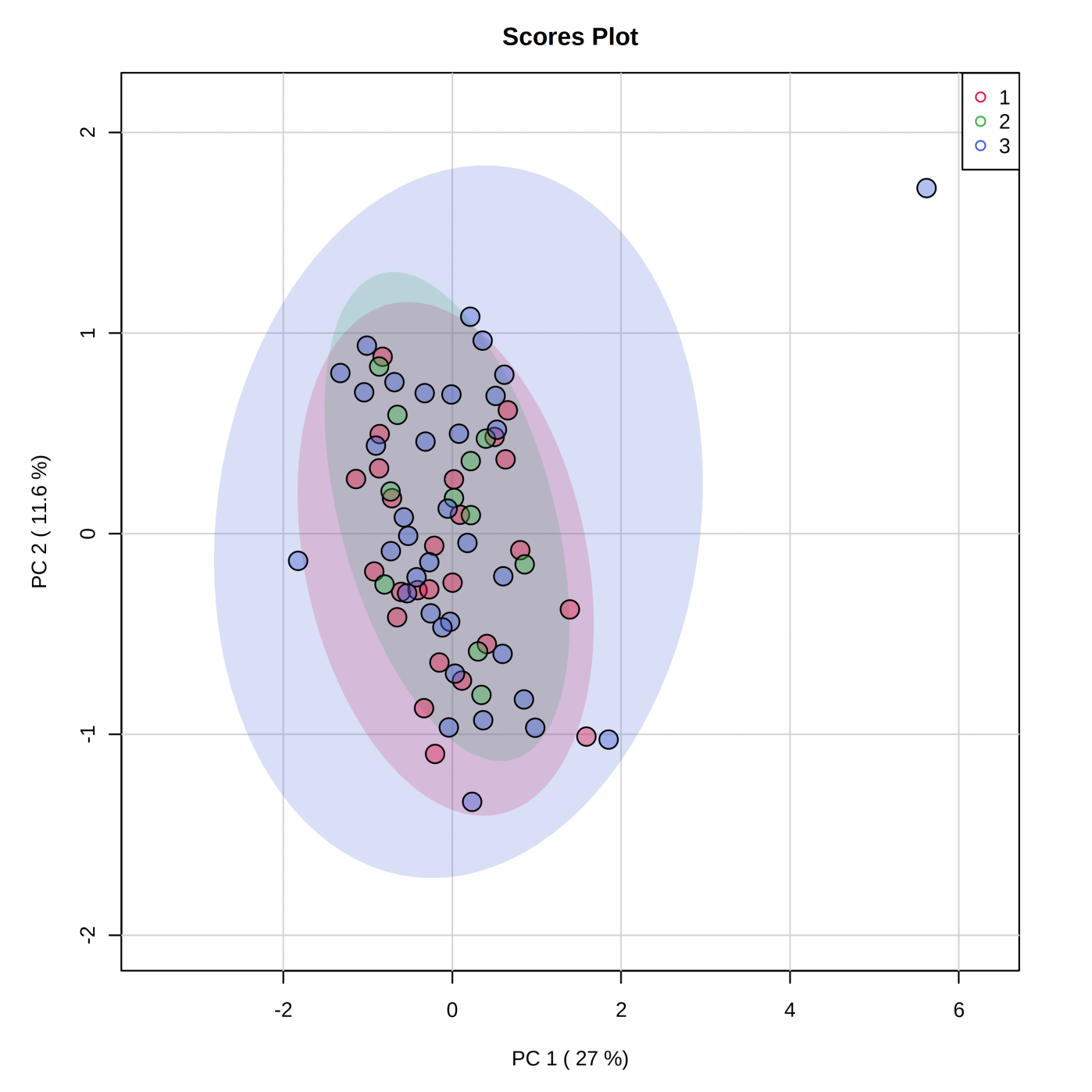


corresponding Biplot (arrows showing importance of parameters)


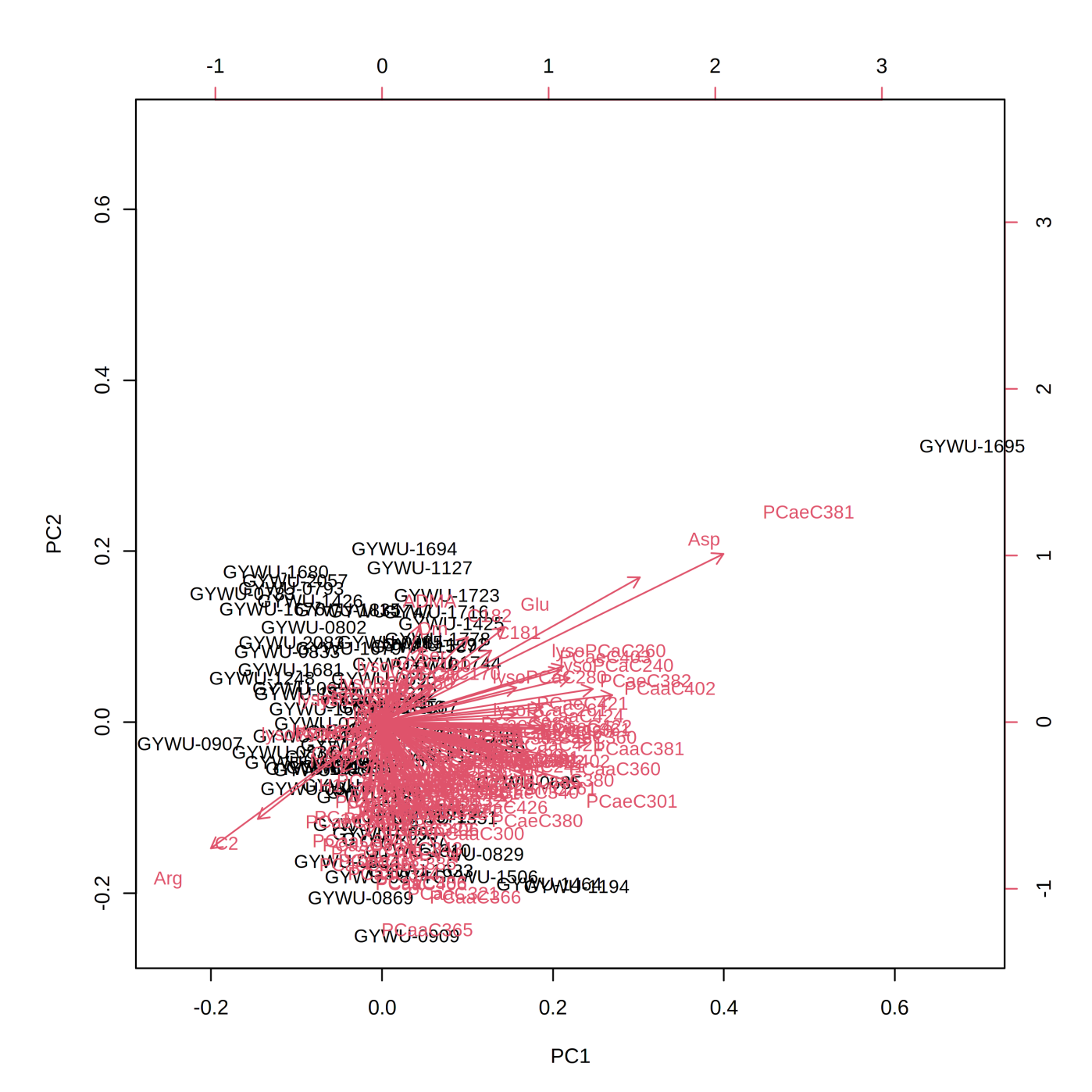


**diagnosis**

PCA analysis (including 95% confidence regions): (1=Pheo, 2=PGL, 3= control) of 130 metabolites (log normalization)


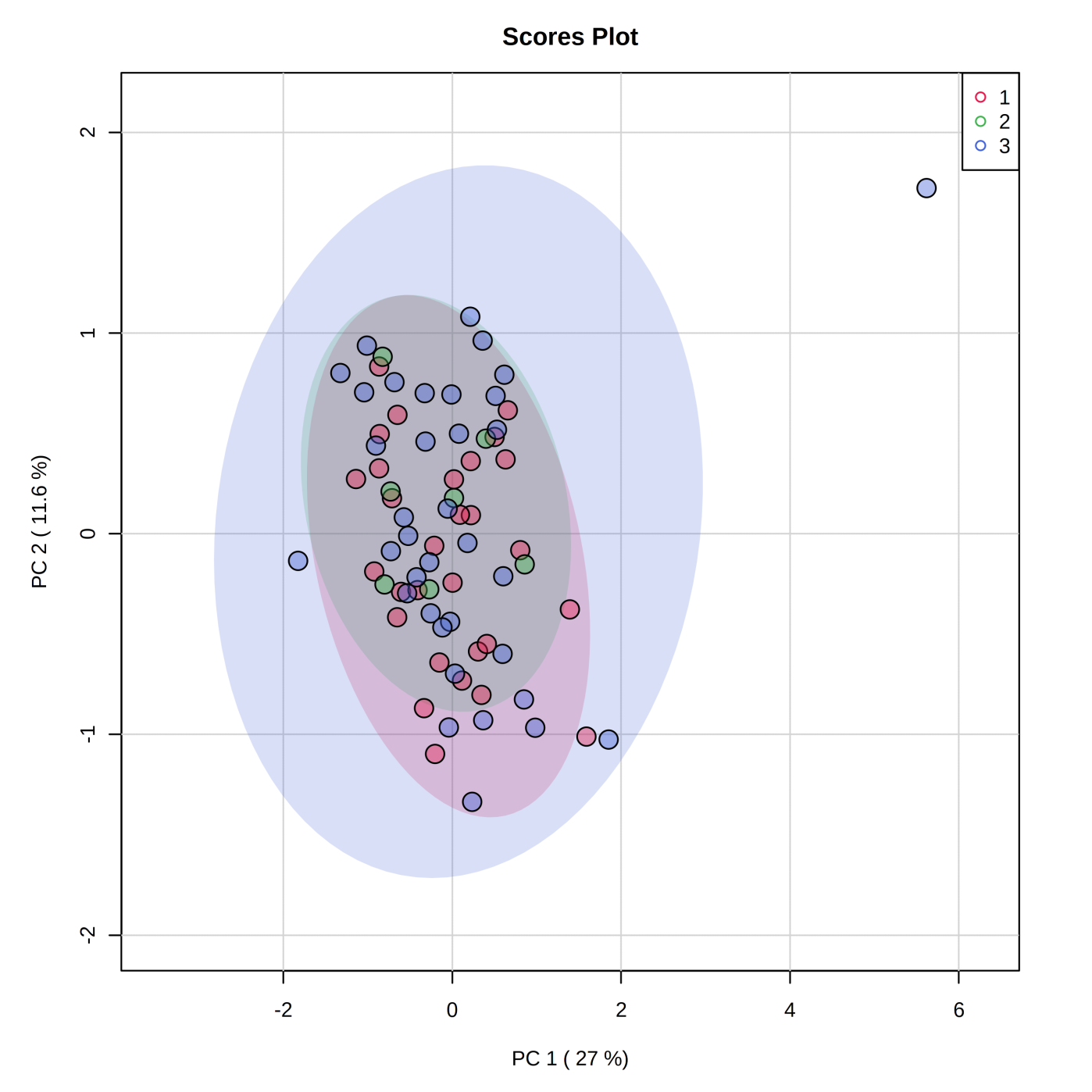


corresponding Biplot (arrows showing importance of parameters)


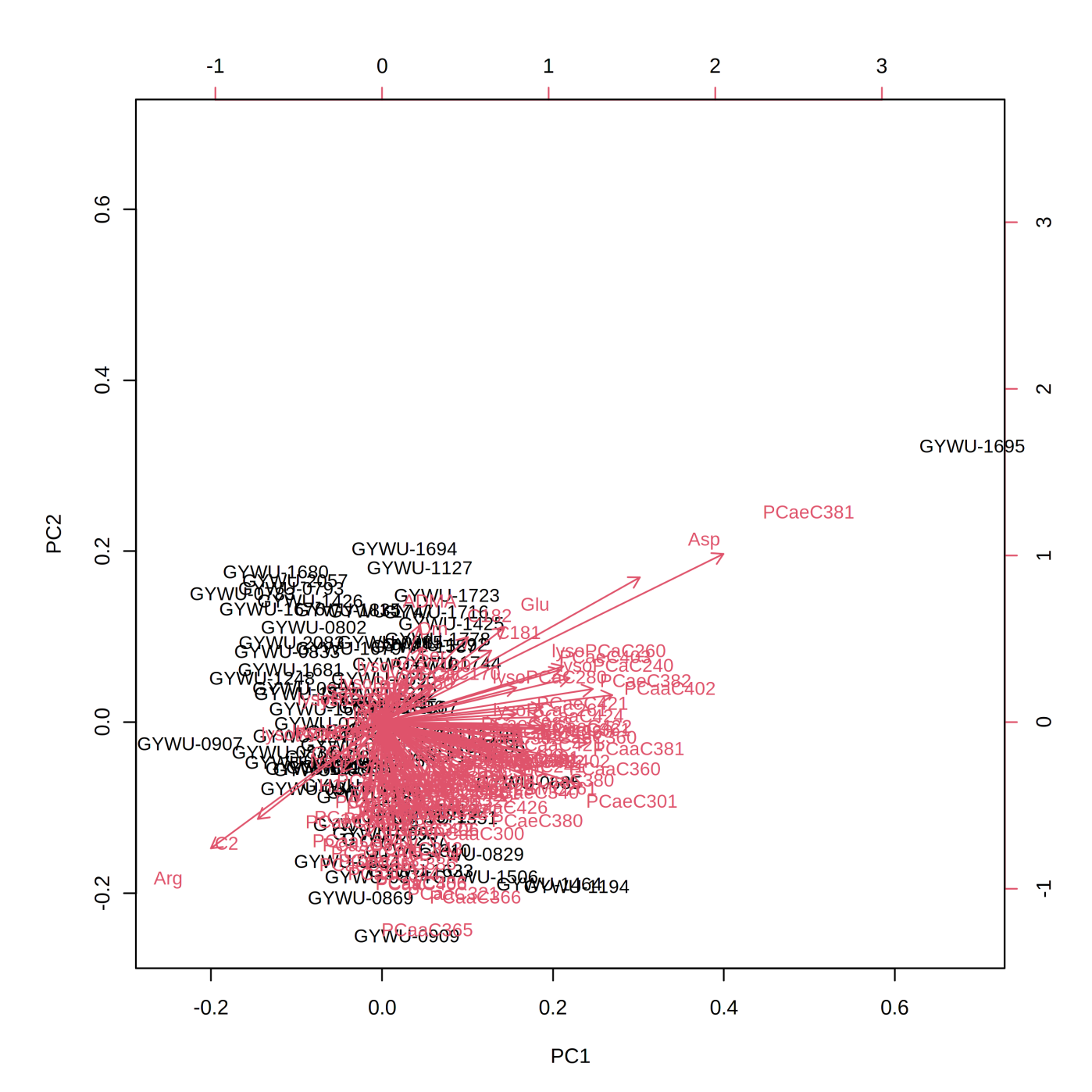


Wilcoxon signed-rank test for selected variables (GBM)

| Variable | Class | Mean | Median | SD | P value (Wilcoxon rank sum test) |
| --- | --- | --- | --- | --- | --- |
| H1 | 1 | 5062 | 4844 | 1731 | **0.01802** |
|  | 2 | 4343 | 4216 | 1024 |  |
| Val | 1 | 227 | 233 | 50.6 | 0.4109 |
|  | 2 | 241 | 234 | 53.5 |  |
| Ala | 1 | 352 | 344 | 97 | 0.09331 |
|  | 2 | 393 | 387 | 106 |  |
| Lys | 1 | 212 | 208 | 46.5 | 0.3858 |
|  | 2 | 223 | 220 | 48.5 |  |
| Gly | 1 | 247 | 225 | 76.7 | 0.0818 |
|  | 2 | 224 | 205 | 87.3 |  |
| PC.aa.C36.2 | 1 | 202 | 204 | 39.1 | 0.6044 |
|  | 2 | 208 | 200 | 41.7 |  |
| PC.aa.C34.2 | 1 | 414 | 401 | 81.8 | 0.4437 |
|  | 2 | 404 | 386 | 90.3 |  |
| Gln | 1 | 520 | 535 | 107 | 0.5849 |
|  | 2 | 537 | 536 | 128 |  |
| PC.aa.C34.1 | 1 | 247 | 234 | 50.3 | 0.727 |
|  | 2 | 251 | 225 | 77.9 |  |

**FDR corrected p-values (q-values) (PPGL vs. control, BMI subgroup <=25, PPGL=22,control=15)**

|  | PPGL vs. control | | **BMI subgroup <=25**, PPGL=22,control=15 | |
| --- | --- | --- | --- | --- |
| analytes | p-value | q-value | p-value | q-value |
| C0 | 0,224 | 0,959 | 0,551 | 1 |
| C2 | 0,475 | 0,959 | 0,939 | 1 |
| C141 | 0,392 | 0,959 | 0,748 | 1 |
| C142 | 0,404 | 0,959 | 0,614 | 1 |
| C181 | 0,677 | 0,959 | 0,119 | 1 |
| C182 | 0,64 | 0,959 | 0,135 | 1 |
| Ala | 0,092 | 0,959 | 0,435 | 1 |
| Arg | 0,765 | 0,959 | 0,658 | 1 |
| Asn | 0,171 | 0,959 | 0,593 | 1 |
| Asp | 0,079 | 0,959 | 0,334 | 1 |
| Cit | 0,551 | 0,959 | 0,453 | 1 |
| Gln | 0,581 | 0,959 | 0,152 | 1 |
| Glu | 0,414 | 0,959 | 0,202 | 1 |
| Gly | 0,081 | 0,959 | 0,070 | 1 |
| His | 0,004 | 0,520 | 0,006 | 0,390 |
| Ile | 0,879 | 0,966 | 0,143 | 1 |
| Leu | 0,532 | 0,959 | 0,435 | 1 |
| Lys | 0,383 | 0,959 | 0,658 | 1 |
| Met | 0,151 | 0,959 | 0,593 | 1 |
| Orn | 0,086 | 0,959 | 0,963 | 1 |
| Phe | 0,471 | 0,959 | 0,939 | 1 |
| Pro | 0,398 | 0,959 | 0,843 | 1 |
| Ser | 0,656 | 0,959 | 0,867 | 1 |
| Thr | 0,008 | 0,520 | 0,135 | 1 |
| Trp | 0,313 | 0,959 | 0,867 | 1 |
| Tyr | 0,175 | 0,959 | 0,453 | 1 |
| Val | 0,408 | 0,959 | 0,748 | 1 |
| ADMA | 0,062 | 0,959 | 0,237 | 1 |
| Creatinine | 0,464 | 0,959 | 0,249 | 1 |
| Kynurenine | 0,585 | 0,959 | 0,891 | 1 |
| Sarcosine | 0,322 | 0,959 | 0,491 | 1 |
| SDMA | 0,51 | 0,959 | 0,703 | 1 |
| lysoPCaC160 | 0,454 | 0,959 | 0,703 | 1 |
| lysoPCaC161 | 0,371 | 0,959 | 0,511 | 1 |
| lysoPCaC170 | 0,727 | 0,959 | 0,867 | 1 |
| lysoPCaC180 | 0,319 | 0,959 | 0,703 | 1 |
| lysoPCaC181 | 0,853 | 0,966 | 0,819 | 1 |
| lysoPCaC182 | 0,817 | 0,966 | 0,680 | 1 |
| lysoPCaC203 | 0,796 | 0,959 | 0,680 | 1 |
| lysoPCaC204 | 0,083 | 0,959 | 0,052 | 1 |
| lysoPCaC240 | 0,689 | 0,959 | 0,843 | 1 |
| lysoPCaC260 | 0,327 | 0,959 | 0,867 | 1 |
| lysoPCaC261 | 0,791 | 0,959 | 0,319 | 1 |
| lysoPCaC280 | 0,044 | 0,959 | 0,061 | 1 |
| lysoPCaC281 | 0,408 | 0,959 | 0,891 | 1 |
| PCaaC281 | 0,892 | 0,966 | 0,213 | 1 |
| PCaaC300 | 0,796 | 0,959 | 0,417 | 1 |
| PCaaC320 | 0,411 | 0,959 | 0,915 | 1 |
| PCaaC321 | 0,848 | 0,966 | 0,658 | 1 |
| PCaaC322 | 0,857 | 0,966 | 0,658 | 1 |
| PCaaC323 | 0,499 | 0,959 | 0,636 | 1 |
| PCaaC341 | 0,669 | 0,959 | 0,891 | 1 |
| PCaaC342 | 0,44 | 0,959 | 0,963 | 1 |
| PCaaC343 | 0,8 | 0,959 | 0,572 | 1 |
| PCaaC344 | 0,933 | 0,978 | 0,658 | 1 |
| PCaaC360 | 0,401 | 0,959 | 0,276 | 1 |
| PCaaC361 | 0,506 | 0,959 | 0,572 | 1 |
| PCaaC362 | 0,6 | 0,959 | 0,725 | 1 |
| PCaaC363 | 0,566 | 0,959 | 0,891 | 1 |
| PCaaC364 | 0,295 | 0,959 | 0,572 | 1 |
| analytes | p-value | q-value | p-value | q-value |
| PCaaC365 | 0,955 | 0,978 | 0,725 | 1 |
| PCaaC366 | 0,562 | 0,959 | 0,262 | 1 |
| PCaaC380 | 0,569 | 0,959 | 0,435 | 1 |
| PCaaC381 | 0,66 | 0,959 | 0,939 | 1 |
| PCaaC383 | 0,866 | 0,966 | 0,891 | 1 |
| PCaaC384 | 0,267 | 0,959 | 0,383 | 1 |
| PCaaC385 | 0,241 | 0,959 | 0,963 | 1 |
| PCaaC386 | 0,644 | 0,959 | 0,400 | 1 |
| PCaaC402 | 0,748 | 0,959 | 0,939 | 1 |
| PCaaC403 | 0,892 | 0,966 | 0,891 | 1 |
| PCaaC404 | 0,919 | 0,978 | 0,593 | 1 |
| PCaaC405 | 0,499 | 0,959 | 0,748 | 1 |
| PCaaC406 | 0,87 | 0,966 | 0,491 | 1 |
| PCaaC420 | 0,593 | 0,959 | 0,819 | 1 |
| PCaaC421 | 0,973 | 0,978 | 0,417 | 1 |
| PCaaC422 | 0,796 | 0,959 | 0,891 | 1 |
| PCaaC424 | 0,604 | 0,959 | 0,636 | 1 |
| PCaaC425 | 0,719 | 0,959 | 0,963 | 1 |
| PCaaC426 | 0,787 | 0,959 | 0,290 | 1 |
| PCaeC300 | 0,371 | 0,959 | 0,105 | 1 |
| PCaeC301 | 0,804 | 0,959 | 0,939 | 1 |
| PCaeC302 | 0,681 | 0,959 | 1 | 1 |
| PCaeC321 | 0,955 | 0,978 | 0,127 | 1 |
| PCaeC322 | 0,636 | 0,959 | 0,435 | 1 |
| PCaeC340 | 0,731 | 0,959 | 0,843 | 1 |
| PCaeC341 | 0,566 | 0,959 | 0,843 | 1 |
| PCaeC342 | 0,421 | 0,959 | 0,511 | 1 |
| PCaeC343 | 0,547 | 0,959 | 0,703 | 1 |
| PCaeC360 | 0,973 | 0,978 | 0,867 | 1 |
| PCaeC361 | 0,562 | 0,959 | 0,843 | 1 |
| PCaeC362 | 0,66 | 0,959 | 0,963 | 1 |
| PCaeC363 | 0,213 | 0,959 | 0,119 | 1 |
| PCaeC364 | 0,875 | 0,966 | 0,319 | 1 |
| PCaeC365 | 0,558 | 0,959 | 0,680 | 1 |
| PCaeC380 | 0,66 | 0,959 | 0,262 | 1 |
| PCaeC381 | 0,188 | 0,959 | 0,334 | 1 |
| PCaeC382 | 0,765 | 0,959 | 0,572 | 1 |
| PCaeC383 | 0,761 | 0,959 | 0,636 | 1 |
| PCaeC384 | 0,424 | 0,959 | 0,636 | 1 |
| PCaeC385 | 0,74 | 0,959 | 0,511 | 1 |
| PCaeC386 | 0,978 | 0,978 | 0,400 | 1 |
| PCaeC401 | 0,386 | 0,959 | 1 | 1 |
| PCaeC402 | 0,55 | 0,959 | 0,614 | 1 |
| PCaeC403 | 0,951 | 0,978 | 0,350 | 1 |
| PCaeC404 | 0,765 | 0,959 | 0,703 | 1 |
| PCaeC405 | 0,395 | 0,959 | 0,819 | 1 |
| PCaeC406 | 0,624 | 0,959 | 0,400 | 1 |
| PCaeC421 | 0,636 | 0,959 | 0,748 | 1 |
| PCaeC422 | 0,804 | 0,959 | 0,939 | 1 |
| PCaeC423 | 0,901 | 0,968 | 0,703 | 1 |
| PCaeC424 | 0,748 | 0,959 | 0,636 | 1 |
| PCaeC425 | 0,551 | 0,959 | 0,988 | 1 |
| PCaeC444 | 0,389 | 0,959 | 0,152 | 1 |
| PCaeC445 | 0,22 | 0,959 | 0,988 | 1 |
| PCaeC446 | 0,303 | 0,959 | 0,748 | 1 |
| SMOHC141 | 0,237 | 0,959 | 0,725 | 1 |
| SMOHC161 | 0,224 | 0,959 | 0,867 | 1 |
| SMOHC221 | 0,207 | 0,959 | 0,703 | 1 |
| SMOHC222 | 0,146 | 0,959 | 0,843 | 1 |
| SMOHC241 | 0,875 | 0,966 | 0,680 | 1 |
| SMC160 | 0,258 | 0,959 | 0,915 | 1 |
| SMC161 | 0,562 | 0,959 | 0,891 | 1 |
| SMC180 | 0,33 | 0,959 | 0,703 | 1 |
| SMC181 | 0,215 | 0,959 | 0,249 | 1 |
| analytes | p-value | q-value | p-value | q-value |
| SMC202 | 0,517 | 0,959 | 0,636 | 1 |
| SMC240 | 0,62 | 0,959 | 0,988 | 1 |
| SMC241 | 0,562 | 0,959 | 0,725 | 1 |
| SMC260 | 0,946 | 0,978 | 0,963 | 1 |
| SMC261 | 0,398 | 0,959 | 0,939 | 1 |
| H1 | 0,018 | 0,780 | 0,003 | 0,390 |

**FDR corrected p-values (q-values) (BMI subgroup >25, PPGL=12, control=20)**

|  | BMI subgroup >25, PPGL=12,control=20 | |
| --- | --- | --- |
| analytes | p-value | q-value |
| C0 | 0,272 | 0,977 |
| C2 | 0,552 | 0,977 |
| C141 | 0,659 | 0,977 |
| C142 | 0,924 | 0,977 |
| C181 | 0,048 | 0,78 |
| C182 | 0,044 | 0,78 |
| Ala | 0,083 | 0,977 |
| Arg | 0,346 | 0,977 |
| Asn | 0,289 | 0,977 |
| Asp | 0,307 | 0,977 |
| Cit | 0,578 | 0,977 |
| Gln | 0,744 | 0,977 |
| Glu | 0,076 | 0,977 |
| Gly | 0,632 | 0,977 |
| His | 0,036 | 0,780 |
| Ile | 0,307 | 0,977 |
| Leu | 0,224 | 0,977 |
| Lys | 0,070 | 0,977 |
| Met | 0,170 | 0,977 |
| Orn | 0,036 | 0,780 |
| Phe | 0,431 | 0,977 |
| Pro | 0,744 | 0,977 |
| Ser | 0,604 | 0,977 |
| Thr | 0,036 | 0,780 |
| Trp | 0,170 | 0,977 |
| Tyr | 0,774 | 0,977 |
| Val | 0,346 | 0,977 |
| ADMA | 0,239 | 0,977 |
| Creatinine | 0,659 | 0,977 |
| Kynurenine | 0,552 | 0,977 |
| Sarcosine | 0,387 | 0,977 |
| SDMA | 0,716 | 0,977 |
| lysoPCaC160 | 0,366 | 0,977 |
| lysoPCaC161 | 0,326 | 0,977 |
| lysoPCaC170 | 0,716 | 0,977 |
| lysoPCaC180 | 0,195 | 0,977 |
| lysoPCaC181 | 0,224 | 0,977 |
| lysoPCaC182 | 0,501 | 0,977 |
| lysoPCaC203 | 0,716 | 0,977 |
| lysoPCaC204 | 0,924 | 0,977 |
| lysoPCaC240 | 0,893 | 0,977 |
| lysoPCaC260 | 0,632 | 0,977 |
| lysoPCaC261 | 0,346 | 0,977 |
| lysoPCaC280 | 0,326 | 0,977 |
| lysoPCaC281 | 0,454 | 0,977 |
| analytes | p-value | q-value |
| PCaaC281 | 0,239 | 0,977 |
| PCaaC300 | 1 | 1 |
| PCaaC320 | 0,454 | 0,977 |
| PCaaC321 | 0,863 | 0,977 |
| PCaaC322 | 0,893 | 0,977 |
| PCaaC323 | 0,803 | 0,977 |
| PCaaC341 | 0,744 | 0,977 |
| PCaaC342 | 0,158 | 0,977 |
| PCaaC343 | 0,863 | 0,977 |
| PCaaC344 | 0,985 | 1 |
| PCaaC360 | 0,477 | 0,977 |
| PCaaC361 | 0,578 | 0,977 |
| PCaaC362 | 0,774 | 0,977 |
| PCaaC363 | 0,387 | 0,977 |
| PCaaC364 | 0,454 | 0,977 |
| PCaaC365 | 0,924 | 0,977 |
| PCaaC366 | 0,833 | 0,977 |
| PCaaC380 | 0,954 | 0,984 |
| PCaaC381 | 0,924 | 0,977 |
| PCaaC383 | 0,659 | 0,977 |
| PCaaC384 | 0,803 | 0,977 |
| PCaaC385 | 0,387 | 0,977 |
| PCaaC386 | 0,146 | 0,977 |
| PCaaC402 | 0,893 | 0,977 |
| PCaaC403 | 0,632 | 0,977 |
| PCaaC404 | 0,893 | 0,977 |
| PCaaC405 | 0,774 | 0,977 |
| PCaaC406 | 0,744 | 0,977 |
| PCaaC420 | 0,863 | 0,977 |
| PCaaC421 | 0,744 | 0,977 |
| PCaaC422 | 1 | 1 |
| PCaaC424 | 0,272 | 0,977 |
| PCaaC425 | 0,387 | 0,977 |
| PCaaC426 | 0,833 | 0,977 |
| PCaeC300 | 0,744 | 0,977 |
| PCaeC301 | 0,552 | 0,977 |
| PCaeC302 | 0,552 | 0,977 |
| PCaeC321 | 0,632 | 0,977 |
| PCaeC322 | 0,863 | 0,977 |
| PCaeC340 | 0,833 | 0,977 |
| PCaeC341 | 0,501 | 0,977 |
| PCaeC342 | 0,387 | 0,977 |
| PCaeC343 | 0,307 | 0,977 |
| PCaeC360 | 0,604 | 0,977 |
| PCaeC361 | 0,632 | 0,977 |
| PCaeC362 | 1 | 1 |
| PCaeC363 | 0,744 | 0,977 |
| PCaeC364 | 0,431 | 0,977 |
| PCaeC365 | 0,526 | 0,977 |
| PCaeC380 | 0,893 | 0,977 |
| PCaeC381 | 0,744 | 0,977 |
| PCaeC382 | 0,863 | 0,977 |
| PCaeC383 | 0,632 | 0,977 |
| PCaeC384 | 0,307 | 0,977 |
| PCaeC385 | 0,431 | 0,977 |
| PCaeC386 | 0,632 | 0,977 |
| PCaeC401 | 0,526 | 0,977 |
| PCaeC402 | 0,954 | 0,984 |
| PCaeC403 | 0,744 | 0,977 |
| PCaeC404 | 0,924 | 0,977 |
| PCaeC405 | 0,604 | 0,977 |
| PCaeC406 | 0,346 | 0,977 |
| PCaeC421 | 0,863 | 0,977 |
| PCaeC422 | 0,833 | 0,977 |
| analytes | p-value | q-value |
| PCaeC423 | 0,774 | 0,977 |
| PCaeC424 | 0,744 | 0,977 |
| PCaeC425 | 0,833 | 0,977 |
| PCaeC444 | 0,659 | 0,977 |
| PCaeC445 | 0,659 | 0,977 |
| PCaeC446 | 0,954 | 0,984 |
| SMOHC141 | 0,04 | 0,780 |
| SMOHC161 | 0,099 | 0,977 |
| SMOHC221 | 0,048 | 0,780 |
| SMOHC222 | 0,036 | 0,780 |
| SMOHC241 | 0,774 | 0,977 |
| SMC160 | 0,17 | 0,977 |
| SMC161 | 0,307 | 0,977 |
| SMC180 | 0,17 | 0,977 |
| SMC181 | 0,578 | 0,977 |
| SMC202 | 0,632 | 0,977 |
| SMC240 | 0,454 | 0,977 |
| SMC241 | 0,924 | 0,977 |
| SMC260 | 0,893 | 0,977 |
| SMC261 | 0,501 | 0,977 |
| H1 | 0,431 | 0,977 |

**FDR corrected p-values (q-values) (subgroup male)**

|  | subgroup male | |
| --- | --- | --- |
| analytes | p-value | q-value |
| C0 | 0,686 | 0,883 |
| C2 | 0,583 | 0,833 |
| C141 | 0,435 | 0,794 |
| C142 | 0,488 | 0,802 |
| C181 | 0,488 | 0,802 |
| C182 | 0,470 | 0,794 |
| Ala | 0,246 | 0,780 |
| Arg | 1 | 1 |
| Asn | 0,311 | 0,780 |
| Asp | 0,163 | 0,780 |
| Cit | 0,284 | 0,780 |
| Gln | 0,212 | 0,780 |
| Glu | 0,908 | 0,960 |
| Gly | 0,234 | 0,780 |
| His | 0,311 | 0,780 |
| Ile | 0,665 | 0,873 |
| Leu | 0,729 | 0,911 |
| Lys | 0,506 | 0,802 |
| Met | 0,583 | 0,833 |
| Orn | 0,163 | 0,780 |
| Phe | 0,339 | 0,780 |
| Pro | 0,154 | 0,780 |
| Ser | 0,271 | 0,780 |
| Thr | 0,008 | 0,780 |
| Trp | 0,258 | 0,780 |
| Tyr | 0,354 | 0,780 |
| Val | 0,470 | 0,794 |
| ADMA | 0,297 | 0,780 |
| Creatinine | 0,181 | 0,780 |
| Kynurenine | 0,885 | 0,959 |
| analytes | p-value | q-value |
| Sarcosine | 0,103 | 0,780 |
| SDMA | 0,563 | 0,832 |
| lysoPCaC160 | 0,246 | 0,780 |
| lysoPCaC161 | 0,025 | 0,780 |
| lysoPCaC170 | 0,665 | 0,873 |
| lysoPCaC180 | 0,544 | 0,832 |
| lysoPCaC181 | 0,258 | 0,780 |
| lysoPCaC182 | 0,452 | 0,794 |
| lysoPCaC203 | 0,339 | 0,780 |
| lysoPCaC204 | 0,708 | 0,902 |
| lysoPCaC240 | 0,418 | 0,794 |
| lysoPCaC260 | 0,840 | 0,950 |
| lysoPCaC261 | 0,435 | 0,794 |
| lysoPCaC280 | 0,435 | 0,794 |
| lysoPCaC281 | 0,085 | 0,780 |
| PCaaC281 | 0,354 | 0,780 |
| PCaaC300 | 0,686 | 0,883 |
| PCaaC320 | 0,370 | 0,782 |
| PCaaC321 | 0,297 | 0,780 |
| PCaaC322 | 0,506 | 0,802 |
| PCaaC323 | 0,418 | 0,794 |
| PCaaC341 | 0,563 | 0,832 |
| PCaaC342 | 0,603 | 0,852 |
| PCaaC343 | 0,154 | 0,780 |
| PCaaC344 | 0,370 | 0,782 |
| PCaaC360 | 0,908 | 0,960 |
| PCaaC361 | 0,665 | 0,873 |
| PCaaC362 | 0,544 | 0,832 |
| PCaaC363 | 0,583 | 0,833 |
| PCaaC364 | 0,470 | 0,794 |
| PCaaC365 | 0,644 | 0,872 |
| PCaaC366 | 0,624 | 0,872 |
| PCaaC380 | 0,506 | 0,802 |
| PCaaC381 | 0,325 | 0,780 |
| PCaaC383 | 0,840 | 0,950 |
| PCaaC384 | 0,773 | 0,922 |
| PCaaC385 | 0,863 | 0,951 |
| PCaaC386 | 0,751 | 0,921 |
| PCaaC402 | 0,154 | 0,780 |
| PCaaC403 | 0,773 | 0,922 |
| PCaaC404 | 0,817 | 0,950 |
| PCaaC405 | 0,931 | 0,968 |
| PCaaC406 | 0,954 | 0,984 |
| PCaaC420 | 0,154 | 0,780 |
| PCaaC421 | 0,435 | 0,794 |
| PCaaC422 | 0,116 | 0,780 |
| PCaaC424 | 0,103 | 0,780 |
| PCaaC425 | 0,751 | 0,921 |
| PCaaC426 | 0,885 | 0,959 |
| PCaeC300 | 0,840 | 0,950 |
| PCaeC301 | 0,908 | 0,960 |
| PCaeC302 | 0,040 | 0,780 |
| PCaeC321 | 0,563 | 0,832 |
| PCaeC322 | 0,977 | 0,992 |
| PCaeC340 | 0,385 | 0,782 |
| PCaeC341 | 0,354 | 0,780 |
| PCaeC342 | 0,863 | 0,951 |
| PCaeC343 | 0,729 | 0,911 |
| PCaeC360 | 1 | 1 |
| PCaeC361 | 0,191 | 0,780 |
| PCaeC362 | 0,297 | 0,780 |
| PCaeC363 | 0,325 | 0,780 |
| PCaeC364 | 0,977 | 0,992 |
| PCaeC365 | 0,817 | 0,950 |
| analytes | p-value | q-value |
| PCaeC380 | 0,773 | 0,922 |
| PCaeC381 | 0,544 | 0,832 |
| PCaeC382 | 0,311 | 0,780 |
| PCaeC383 | 0,212 | 0,780 |
| PCaeC384 | 0,470 | 0,794 |
| PCaeC385 | 0,644 | 0,872 |
| PCaeC386 | 0,644 | 0,872 |
| PCaeC401 | 0,234 | 0,780 |
| PCaeC402 | 0,418 | 0,794 |
| PCaeC403 | 0,172 | 0,780 |
| PCaeC404 | 0,271 | 0,780 |
| PCaeC405 | 0,146 | 0,780 |
| PCaeC406 | 0,339 | 0,780 |
| PCaeC421 | 0,325 | 0,780 |
| PCaeC422 | 0,246 | 0,780 |
| PCaeC423 | 0,385 | 0,782 |
| PCaeC424 | 0,258 | 0,780 |
| PCaeC425 | 0,258 | 0,780 |
| PCaeC444 | 0,931 | 0,968 |
| PCaeC445 | 0,271 | 0,780 |
| PCaeC446 | 0,223 | 0,780 |
| SMOHC141 | 0,040 | 0,780 |
| SMOHC161 | 0,070 | 0,780 |
| SMOHC221 | 0,172 | 0,780 |
| SMOHC222 | 0,103 | 0,780 |
| SMOHC241 | 0,258 | 0,780 |
| SMC160 | 0,130 | 0,780 |
| SMC161 | 0,246 | 0,780 |
| SMC180 | 0,452 | 0,794 |
| SMC181 | 0,311 | 0,780 |
| SMC202 | 0,840 | 0,950 |
| SMC240 | 0,201 | 0,780 |
| SMC241 | 0,863 | 0,951 |
| SMC260 | 0,385 | 0,782 |
| SMC261 | 0,325 | 0,780 |
| H1 | 0,050 | 0,780 |

**FDR corrected p-values (q-values) (subgroup female)**

|  | subgroup male | |
| --- | --- | --- |
| analytes | p-value | q-value |
| C0 | 0,160 | 0,930 |
| C2 | 0,708 | 0,930 |
| C141 | 0,563 | 0,930 |
| C142 | 0,540 | 0,930 |
| C181 | 0,946 | 0,968 |
| C182 | 1,000 | 1 |
| Ala | 0,290 | 0,930 |
| Arg | 0,786 | 0,934 |
| Asn | 0,375 | 0,930 |
| Asp | 0,259 | 0,930 |
| Cit | 0,812 | 0,934 |
| Gln | 0,708 | 0,930 |
| Glu | 0,306 | 0,930 |
| Gly | 0,182 | 0,930 |
| His | 0,001 | 0,130 |
| Ile | 0,734 | 0,934 |
| Leu | 0,734 | 0,934 |
| Lys | 0,540 | 0,930 |
| analytes | p-value | q-value |
| Met | 0,131 | 0,930 |
| Orn | 0,357 | 0,930 |
| Phe | 0,865 | 0,953 |
| Pro | 0,760 | 0,934 |
| Ser | 0,610 | 0,930 |
| Thr | 0,290 | 0,930 |
| Trp | 0,734 | 0,934 |
| Tyr | 0,433 | 0,930 |
| Val | 0,634 | 0,930 |
| ADMA | 0,122 | 0,930 |
| Creatinine | 0,919 | 0,963 |
| Kynurenine | 0,339 | 0,930 |
| Sarcosine | 0,683 | 0,930 |
| SDMA | 0,708 | 0,930 |
| lysoPCaC160 | 0,838 | 0,947 |
| lysoPCaC161 | 0,306 | 0,930 |
| lysoPCaC170 | 0,919 | 0,963 |
| lysoPCaC180 | 0,586 | 0,930 |
| lysoPCaC181 | 0,231 | 0,930 |
| lysoPCaC182 | 0,306 | 0,930 |
| lysoPCaC203 | 0,092 | 0,930 |
| lysoPCaC204 | 0,006 | 0,390 |
| lysoPCaC240 | 0,193 | 0,930 |
| lysoPCaC260 | 0,114 | 0,930 |
| lysoPCaC261 | 0,786 | 0,934 |
| lysoPCaC280 | 0,067 | 0,930 |
| lysoPCaC281 | 0,413 | 0,930 |
| PCaaC281 | 0,131 | 0,930 |
| PCaaC300 | 0,838 | 0,947 |
| PCaaC320 | 0,658 | 0,930 |
| PCaaC321 | 0,433 | 0,930 |
| PCaaC322 | 0,708 | 0,930 |
| PCaaC323 | 0,892 | 0,963 |
| PCaaC341 | 0,231 | 0,930 |
| PCaaC342 | 0,708 | 0,930 |
| PCaaC343 | 0,454 | 0,930 |
| PCaaC344 | 0,433 | 0,930 |
| PCaaC360 | 0,150 | 0,930 |
| PCaaC361 | 0,610 | 0,930 |
| PCaaC362 | 0,634 | 0,930 |
| PCaaC363 | 0,274 | 0,930 |
| PCaaC364 | 0,041 | 0,930 |
| PCaaC365 | 0,658 | 0,930 |
| PCaaC366 | 0,786 | 0,934 |
| PCaaC380 | 0,085 | 0,930 |
| PCaaC381 | 0,634 | 0,930 |
| PCaaC383 | 0,610 | 0,930 |
| PCaaC384 | 0,049 | 0,930 |
| PCaaC385 | 0,150 | 0,930 |
| PCaaC386 | 0,812 | 0,934 |
| PCaaC402 | 0,357 | 0,930 |
| PCaaC403 | 0,634 | 0,930 |
| PCaaC404 | 0,634 | 0,930 |
| PCaaC405 | 0,274 | 0,930 |
| PCaaC406 | 0,786 | 0,934 |
| PCaaC420 | 0,375 | 0,930 |
| PCaaC421 | 0,357 | 0,930 |
| PCaaC422 | 0,518 | 0,930 |
| PCaaC424 | 0,394 | 0,930 |
| PCaaC425 | 0,760 | 0,934 |
| PCaaC426 | 0,634 | 0,930 |
| PCaeC300 | 0,140 | 0,930 |
| PCaeC301 | 0,474 | 0,930 |
| PCaeC302 | 0,150 | 0,930 |
| analytes | p-value | q-value |
| PCaeC321 | 0,563 | 0,930 |
| PCaeC322 | 0,245 | 0,930 |
| PCaeC340 | 0,540 | 0,930 |
| PCaeC341 | 0,812 | 0,934 |
| PCaeC342 | 0,433 | 0,930 |
| PCaeC343 | 0,610 | 0,930 |
| PCaeC360 | 1 | 1 |
| PCaeC361 | 0,375 | 0,930 |
| PCaeC362 | 0,610 | 0,930 |
| PCaeC363 | 0,474 | 0,930 |
| PCaeC364 | 0,865 | 0,953 |
| PCaeC365 | 0,708 | 0,930 |
| PCaeC380 | 0,658 | 0,930 |
| PCaeC381 | 0,022 | 0,930 |
| PCaeC382 | 0,170 | 0,930 |
| PCaeC383 | 0,563 | 0,930 |
| PCaeC384 | 0,610 | 0,930 |
| PCaeC385 | 0,919 | 0,963 |
| PCaeC386 | 0,610 | 0,930 |
| PCaeC401 | 0,919 | 0,963 |
| PCaeC402 | 0,892 | 0,963 |
| PCaeC403 | 0,205 | 0,930 |
| PCaeC404 | 0,540 | 0,930 |
| PCaeC405 | 0,708 | 0,930 |
| PCaeC406 | 0,683 | 0,930 |
| PCaeC421 | 0,658 | 0,930 |
| PCaeC422 | 0,394 | 0,930 |
| PCaeC423 | 0,433 | 0,930 |
| PCaeC424 | 0,563 | 0,930 |
| PCaeC425 | 0,683 | 0,930 |
| PCaeC444 | 0,231 | 0,930 |
| PCaeC445 | 0,563 | 0,930 |
| PCaeC446 | 0,812 | 0,934 |
| SMOHC141 | 0,454 | 0,930 |
| SMOHC161 | 0,865 | 0,953 |
| SMOHC221 | 0,946 | 0,968 |
| SMOHC222 | 0,760 | 0,934 |
| SMOHC241 | 0,259 | 0,930 |
| SMC160 | 0,973 | 0,988 |
| SMC161 | 0,683 | 0,930 |
| SMC180 | 0,474 | 0,930 |
| SMC181 | 0,413 | 0,930 |
| SMC202 | 0,658 | 0,930 |
| SMC240 | 0,563 | 0,930 |
| SMC241 | 0,610 | 0,930 |
| SMC260 | 0,394 | 0,930 |
| SMC261 | 0,946 | 0,968 |
| H1 | 0,218 | 0,930 |

**FDR corrected p-values (q-values) (subgroup adrenergic)**

|  | subgroup adrenergic | |
| --- | --- | --- |
| analytes | p-value | q-value |
| C0 | 0,520 | 0,991 |
| C2 | 0,724 | 0,991 |
| C141 | 1,000 | 1,000 |
| C142 | 0,663 | 0,991 |
| C181 | 0,254 | 0,991 |
| C182 | 0,819 | 0,991 |
| analytes | p-value | q-value |
| Ala | 0,395 | 0,991 |
| Arg | 0,885 | 0,991 |
| Asn | 0,407 | 0,991 |
| Asp | 0,494 | 0,991 |
| Cit | 0,820 | 0,991 |
| Gln | 0,740 | 0,991 |
| Glu | 0,803 | 0,991 |
| Gly | 0,007 | 0,455 |
| His | 0,004 | 0,455 |
| Ile | 0,384 | 0,991 |
| Leu | 0,885 | 0,991 |
| Lys | 0,967 | 0,991 |
| Met | 0,756 | 0,991 |
| Orn | 0,590 | 0,991 |
| Phe | 0,678 | 0,991 |
| Pro | 0,917 | 0,991 |
| Ser | 0,141 | 0,991 |
| Thr | 0,678 | 0,991 |
| Trp | 0,198 | 0,991 |
| Tyr | 0,983 | 0,991 |
| Val | 0,455 | 0,991 |
| ADMA | 0,468 | 0,991 |
| Creatinine | 0,494 | 0,991 |
| Kynurenine | 0,135 | 0,991 |
| Sarcosine | 0,771 | 0,991 |
| SDMA | 0,290 | 0,991 |
| lysoPCaC160 | 0,590 | 0,991 |
| lysoPCaC161 | 0,271 | 0,991 |
| lysoPCaC170 | 0,576 | 0,991 |
| lysoPCaC180 | 0,740 | 0,991 |
| lysoPCaC181 | 0,213 | 0,991 |
| lysoPCaC182 | 0,507 | 0,991 |
| lysoPCaC203 | 0,152 | 0,991 |
| lysoPCaC204 | 0,019 | 0,823 |
| lysoPCaC240 | 0,604 | 0,991 |
| lysoPCaC260 | 0,272 | 0,991 |
| lysoPCaC261 | 0,395 | 0,991 |
| lysoPCaC280 | 0,026 | 0,845 |
| lysoPCaC281 | 0,967 | 0,991 |
| PCaaC281 | 0,590 | 0,991 |
| PCaaC300 | 0,950 | 0,991 |
| PCaaC320 | 0,983 | 0,991 |
| PCaaC321 | 0,575 | 0,991 |
| PCaaC322 | 0,820 | 0,991 |
| PCaaC323 | 0,868 | 0,991 |
| PCaaC341 | 0,803 | 0,991 |
| PCaaC342 | 0,633 | 0,991 |
| PCaaC343 | 0,756 | 0,991 |
| PCaaC344 | 0,678 | 0,991 |
| PCaaC360 | 0,372 | 0,991 |
| PCaaC361 | 0,237 | 0,991 |
| PCaaC362 | 0,135 | 0,991 |
| PCaaC363 | 0,633 | 0,991 |
| PCaaC364 | 0,967 | 0,991 |
| PCaaC365 | 0,756 | 0,991 |
| PCaaC366 | 0,852 | 0,991 |
| PCaaC380 | 0,407 | 0,991 |
| PCaaC381 | 0,950 | 0,991 |
| PCaaC383 | 0,917 | 0,991 |
| PCaaC384 | 0,917 | 0,991 |
| PCaaC385 | 0,756 | 0,991 |
| PCaaC386 | 0,772 | 0,991 |
| PCaaC402 | 0,443 | 0,991 |
| PCaaC403 | 0,575 | 0,991 |
| analytes | p-value | q-value |
| PCaaC404 | 0,407 | 0,991 |
| PCaaC405 | 0,983 | 0,991 |
| PCaaC406 | 0,372 | 0,991 |
| PCaaC420 | 0,852 | 0,991 |
| PCaaC421 | 0,468 | 0,991 |
| PCaaC422 | 0,431 | 0,991 |
| PCaaC424 | 0,493 | 0,991 |
| PCaaC425 | 0,263 | 0,991 |
| PCaaC426 | 0,372 | 0,991 |
| PCaeC300 | 0,507 | 0,991 |
| PCaeC301 | 0,934 | 0,991 |
| PCaeC302 | 0,885 | 0,991 |
| PCaeC321 | 0,604 | 0,991 |
| PCaeC322 | 0,852 | 0,991 |
| PCaeC340 | 0,709 | 0,991 |
| PCaeC341 | 0,709 | 0,991 |
| PCaeC342 | 0,494 | 0,991 |
| PCaeC343 | 0,419 | 0,991 |
| PCaeC360 | 0,648 | 0,991 |
| PCaeC361 | 0,983 | 0,991 |
| PCaeC362 | 0,694 | 0,991 |
| PCaeC363 | 0,351 | 0,991 |
| PCaeC364 | 0,950 | 0,991 |
| PCaeC365 | 0,756 | 0,991 |
| PCaeC380 | 0,678 | 0,991 |
| PCaeC381 | 0,097 | 0,991 |
| PCaeC382 | 0,419 | 0,991 |
| PCaeC383 | 0,694 | 0,991 |
| PCaeC384 | 0,967 | 0,991 |
| PCaeC385 | 0,868 | 0,991 |
| PCaeC386 | 0,787 | 0,991 |
| PCaeC401 | 0,868 | 0,991 |
| PCaeC402 | 0,884 | 0,991 |
| PCaeC403 | 0,300 | 0,991 |
| PCaeC404 | 0,383 | 0,991 |
| PCaeC405 | 0,590 | 0,991 |
| PCaeC406 | 0,648 | 0,991 |
| PCaeC421 | 0,468 | 0,991 |
| PCaeC422 | 0,852 | 0,991 |
| PCaeC423 | 0,373 | 0,991 |
| PCaeC424 | 0,756 | 0,991 |
| PCaeC425 | 0,443 | 0,991 |
| PCaeC444 | 0,373 | 0,991 |
| PCaeC445 | 0,787 | 0,991 |
| PCaeC446 | 0,934 | 0,991 |
| SMOHC141 | 0,455 | 0,991 |
| SMOHC161 | 0,178 | 0,991 |
| SMOHC221 | 0,507 | 0,991 |
| SMOHC222 | 0,384 | 0,991 |
| SMOHC241 | 0,852 | 0,991 |
| SMC160 | 0,443 | 0,991 |
| SMC161 | 0,917 | 0,991 |
| SMC180 | 0,101 | 0,991 |
| SMC181 | 0,221 | 0,991 |
| SMC202 | 0,547 | 0,991 |
| SMC240 | 0,885 | 0,991 |
| SMC241 | 0,604 | 0,991 |
| SMC260 | 0,967 | 0,991 |
| SMC261 | 0,534 | 0,991 |
| H1 | 0,081 | 0,991 |

**FDR corrected p-values (q-values) (subgroup noradrenergic)**

|  | subgroup noradrenergic | |
| --- | --- | --- |
| analytes | p-value | q-value |
| C0 | 0,042 | 0,927 |
| C2 | 0,285 | 0,927 |
| C141 | 0,296 | 0,927 |
| C142 | 0,174 | 0,927 |
| C181 | 0,178 | 0,927 |
| C182 | 0,420 | 0,927 |
| Ala | 0,122 | 0,927 |
| Arg | 0,900 | 0,965 |
| Asn | 0,013 | 0,845 |
| Asp | 0,116 | 0,927 |
| Cit | 0,706 | 0,927 |
| Gln | 0,406 | 0,927 |
| Glu | 0,372 | 0,927 |
| Gly | 0,725 | 0,927 |
| His | 0,195 | 0,927 |
| Ile | 0,392 | 0,927 |
| Leu | 0,580 | 0,927 |
| Lys | 0,345 | 0,927 |
| Met | 0,152 | 0,927 |
| Orn | 0,110 | 0,927 |
| Phe | 0,505 | 0,927 |
| Pro | 0,237 | 0,927 |
| Ser | 0,624 | 0,927 |
| Thr | 0,002 | 0,260 |
| Trp | 0,725 | 0,927 |
| Tyr | 0,099 | 0,927 |
| Val | 0,633 | 0,927 |
| ADMA | 0,048 | 0,927 |
| Creatinine | 0,840 | 0,964 |
| Kynurenine | 0,669 | 0,927 |
| Sarcosine | 0,128 | 0,927 |
| SDMA | 0,061 | 0,927 |
| lysoPCaC160 | 0,204 | 0,927 |
| lysoPCaC161 | 0,072 | 0,927 |
| lysoPCaC170 | 0,980 | 0,980 |
| lysoPCaC180 | 0,399 | 0,927 |
| lysoPCaC181 | 0,227 | 0,927 |
| lysoPCaC182 | 0,606 | 0,927 |
| lysoPCaC203 | 0,358 | 0,927 |
| lysoPCaC204 | 0,860 | 0,964 |
| lysoPCaC240 | 0,970 | 0,978 |
| lysoPCaC260 | 0,538 | 0,927 |
| lysoPCaC261 | 0,687 | 0,927 |
| lysoPCaC280 | 0,650 | 0,927 |
| lysoPCaC281 | 0,291 | 0,927 |
| PCaaC281 | 0,606 | 0,927 |
| PCaaC300 | 0,725 | 0,927 |
| PCaaC320 | 0,345 | 0,927 |
| PCaaC321 | 0,352 | 0,927 |
| PCaaC322 | 0,669 | 0,927 |
| PCaaC323 | 0,421 | 0,927 |
| PCaaC341 | 0,772 | 0,929 |
| PCaaC342 | 0,268 | 0,927 |
| PCaaC343 | 0,850 | 0,964 |
| PCaaC344 | 0,734 | 0,927 |
| PCaaC360 | 0,687 | 0,927 |
| PCaaC361 | 0,950 | 0,965 |
| analytes | p-value | q-value |
| PCaaC362 | 0,744 | 0,927 |
| PCaaC363 | 0,678 | 0,927 |
| PCaaC364 | 0,302 | 0,927 |
| PCaaC365 | 0,940 | 0,965 |
| PCaaC366 | 0,633 | 0,927 |
| PCaaC380 | 0,950 | 0,965 |
| PCaaC381 | 0,489 | 0,927 |
| PCaaC383 | 0,840 | 0,964 |
| PCaaC384 | 0,199 | 0,927 |
| PCaaC385 | 0,279 | 0,927 |
| PCaaC386 | 0,529 | 0,927 |
| PCaaC402 | 0,263 | 0,927 |
| PCaaC403 | 0,831 | 0,964 |
| PCaaC404 | 0,392 | 0,927 |
| PCaaC405 | 0,308 | 0,927 |
| PCaaC406 | 0,821 | 0,964 |
| PCaaC420 | 0,314 | 0,927 |
| PCaaC421 | 0,563 | 0,927 |
| PCaaC422 | 0,358 | 0,927 |
| PCaaC424 | 0,232 | 0,927 |
| PCaaC425 | 0,633 | 0,927 |
| PCaaC426 | 0,538 | 0,927 |
| PCaeC300 | 0,651 | 0,927 |
| PCaeC301 | 0,860 | 0,964 |
| PCaeC302 | 0,443 | 0,927 |
| PCaeC321 | 0,880 | 0,965 |
| PCaeC322 | 0,333 | 0,927 |
| PCaeC340 | 0,450 | 0,927 |
| PCaeC341 | 0,589 | 0,927 |
| PCaeC342 | 0,930 | 0,965 |
| PCaeC343 | 0,930 | 0,965 |
| PCaeC360 | 0,505 | 0,927 |
| PCaeC361 | 0,414 | 0,927 |
| PCaeC362 | 0,247 | 0,927 |
| PCaeC363 | 0,597 | 0,927 |
| PCaeC364 | 0,763 | 0,927 |
| PCaeC365 | 0,734 | 0,927 |
| PCaeC380 | 0,910 | 0,965 |
| PCaeC381 | 0,753 | 0,927 |
| PCaeC382 | 0,744 | 0,927 |
| PCaeC383 | 0,428 | 0,927 |
| PCaeC384 | 0,314 | 0,927 |
| PCaeC385 | 0,725 | 0,927 |
| PCaeC386 | 0,940 | 0,965 |
| PCaeC401 | 0,170 | 0,927 |
| PCaeC402 | 0,546 | 0,927 |
| PCaeC403 | 0,538 | 0,927 |
| PCaeC404 | 0,263 | 0,927 |
| PCaeC405 | 0,110 | 0,927 |
| PCaeC406 | 0,222 | 0,927 |
| PCaeC421 | 0,232 | 0,927 |
| PCaeC422 | 0,580 | 0,927 |
| PCaeC423 | 0,308 | 0,927 |
| PCaeC424 | 0,473 | 0,927 |
| PCaeC425 | 0,182 | 0,927 |
| PCaeC444 | 0,706 | 0,927 |
| PCaeC445 | 0,174 | 0,927 |
| PCaeC446 | 0,182 | 0,927 |
| SMOHC141 | 0,392 | 0,927 |
| SMOHC161 | 0,763 | 0,927 |
| SMOHC221 | 0,302 | 0,927 |
| SMOHC222 | 0,263 | 0,927 |
| SMOHC241 | 0,753 | 0,927 |
| SMC160 | 0,352 | 0,927 |
| analytes | p-value | q-value |
| SMC161 | 0,443 | 0,927 |
| SMC180 | 0,940 | 0,965 |
| SMC181 | 0,513 | 0,927 |
| SMC202 | 0,880 | 0,965 |
| SMC240 | 0,615 | 0,927 |
| SMC241 | 0,792 | 0,945 |
| SMC260 | 0,940 | 0,965 |
| SMC261 | 0,715 | 0,927 |
| H1 | 0,092 | 0,927 |

**Metabolite data**

| **metabolite** | **class** | **n < LLOQ** | **% < LLOQ** | **LC-MS/MS** | **FIA-MS/MS** | **PPGL - group** | | **control- group** | | **p -value** |  |  |
| --- | --- | --- | --- | --- | --- | --- | --- | --- | --- | --- | --- | --- |
|  |  |  |  |  |  | Median | IQR | Median | IQR |  |  |  |
| C0 | Acylcarnitine | 0 | 0% |  | X | 35,6 | 29,40 - 41,18 | 37,95 | 31,55 - 45,78 | 0,224 |  |  |
| C2 | Acylcarnitine | 1 | 1% |  | X | 5,09 | 3,79 - 7,51 | 5,80 | 4,74 - 7,03 | 0,475 |  |  |
| C3 | Acylcarnitine | 30 | 42% |  | X |  |  |  |  |  |  |  |
| C3-DC (C4-OH) | Acylcarnitine | 68 | 94% |  | X |  |  |  |  |  |  |  |
| C3-OH | Acylcarnitine | 72 | 100% |  | X |  |  |  |  |  |  |  |
| C3:1 | Acylcarnitine | 72 | 100% |  | X |  |  |  |  |  |  |  |
| C4 | Acylcarnitine | 68 | 94% |  | X |  |  |  |  |  |  |  |
| C4:1 | Acylcarnitine | 72 | 100% |  | X |  |  |  |  |  |  |  |
| C5 | Acylcarnitine | 71 | 99% |  | X |  |  |  |  |  |  |  |
| C5-DC (C6-OH) | Acylcarnitine | 71 | 99% |  | X |  |  |  |  |  |  |  |
| C5-M-DC | Acylcarnitine | 71 | 99% |  | X |  |  |  |  |  |  |  |
| C5-OH (C3-DC-M) | Acylcarnitine | 71 | 99% |  | X |  |  |  |  |  |  |  |
| C5:1 | Acylcarnitine | 72 | 100% |  | X |  |  |  |  |  |  |  |
| C5:1-DC | Acylcarnitine | 72 | 100% |  | X |  |  |  |  |  |  |  |
| C6 (C4:1-DC) | Acylcarnitine | 70 | 97% |  | X |  |  |  |  |  |  |  |
| C6:1 | Acylcarnitine | 72 | 100% |  | X |  |  |  |  |  |  |  |
| C7-DC | Acylcarnitine | 71 | 99% |  | X |  |  |  |  |  |  |  |
| C8 | Acylcarnitine | 66 | 92% |  | X |  |  |  |  |  |  |  |
| C9 | Acylcarnitine | 71 | 99% |  | X |  |  |  |  |  |  |  |
| C10 | Acylcarnitine | 61 | 85% |  | X |  |  |  |  |  |  |  |
| C10:1 | Acylcarnitine | 58 | 81% |  | X |  |  |  |  |  |  |  |
| C10:2 | Acylcarnitine | 72 | 100% |  | X |  |  |  |  |  |  |  |
| C12 | Acylcarnitine | 72 | 100% |  | X |  |  |  |  |  |  |  |
| C12-DC | Acylcarnitine | 72 | 100% |  | X |  |  |  |  |  |  |  |
| C12:1 | Acylcarnitine | 29 | 40% |  | X |  |  |  |  |  |  |  |
| C14 | Acylcarnitine | 72 | 100% |  | X |  |  |  |  |  |  |  |
| C14:1 | Acylcarnitine | 10 | 14% |  | X | 0,05 | 0,04 - 0,07 | 0,05 | 0,04 - 0,08 | 0,392 |  |  |
| C14:1-OH | Acylcarnitine | 71 | 99% |  | X |  |  |  |  |  |  |  |
| C14:2 | Acylcarnitine | 28 | 39% |  | X | 0,02 | 0,02 - 0,03 | 0,02 | 0,02 - 0,03 | 0,404 |  |  |
| C14:2-OH | Acylcarnitine | 71 | 99% |  | X |  |  |  |  |  |  |  |
| C16 | Acylcarnitine | 71 | 99% |  | X |  |  |  |  |  |  |  |
| C16-OH | Acylcarnitine | 72 | 100% |  | X |  |  |  |  |  |  |  |
| C16:1 | Acylcarnitine | 70 | 97% |  | X |  |  |  |  |  |  |  |
| C16:1-OH | Acylcarnitine | 70 | 97% |  | X |  |  |  |  |  |  |  |
| C16:2 | Acylcarnitine | 72 | 100% |  | X |  |  |  |  |  |  |  |
| C16:2-OH | Acylcarnitine | 72 | 100% |  | X |  |  |  |  |  |  |  |
| C18 | Acylcarnitine | 71 | 99% |  | X |  |  |  |  |  |  |  |
| C18:1 | Acylcarnitine | 0 | 0% |  | X | 0,11 | 0,09 - 0,14 | 0,11 | 0,09 - 0,15 | 0,677 |  |  |
| C18:1-OH | Acylcarnitine | 72 | 100% |  | X |  |  |  |  |  |  |  |
| C18:2 | Acylcarnitine | 0 | 0% |  | X | 0,03 | 0,03 - 0,05 | 0,03 | 0,03 - 0,05 | 0,640 |  |  |
| Alanine | Amino acids | 0 | 0% | X |  | 343,5 | 278,50 - 404,75 | 387,00 | 311,50 - 485,25 | 0,092 |  |  |
| Arginine | Amino acids | 1 | 1% | X |  | 69,55 | 53,78 - 94,03 | 69,90 | 48,40 - 90,43 | 0,765 |  |  |
| Asparagine | Amino acids | 0 | 0% | X |  | 37,25 | 31,63 - 42,20 | 40,55 | 32,10 - 46,75 | 0,171 |  |  |
| Aspartic Acid | Amino acids | 26 | 36% | X |  | 5,24 | 3,24 - 9,38 | 7,78 | 4,45 - 13,55 | 0,079 |  |  |
| Citrulline | Amino acids | 0 | 0% | X |  | 30,35 | 25,75 - 34,78 | 30,90 | 24,63 - 38,30 | 0,551 |  |  |
| Glutamic acid | Amino acids | 0 | 0% | X |  | 535 | 436,75 - 564,50 | 535,50 | 469,75 - 633,75 | 0,581 |  |  |
| Glutamine | Amino acids | 0 | 0% | X |  | 100,45 | 78,13 - 126,50 | 107,00 | 81,23 - 170,50 | 0,414 |  |  |
| Glycine | Amino acids | 0 | 0% | X |  | 225,00 | 192,75 - 291,50 | 205,00 | 167,00 - 256,75 | 0,081 |  |  |
| Histidine | Amino acids | 0 | 0% | X |  | 75,40 | 61,03 - 87,05 | 86,40 | 75,63 - 96,35 | 0,004 |  |  |
| Isoleucine | Amino acids | 0 | 0% | X |  | 73,55 | 60,53 - 81,00 | 69,20 | 61,08 - 88,68 | 0,879 |  |  |
| Leucine | Amino acids | 0 | 0% | X |  | 131,00 | 113,25 - 157,50 | 135,50 | 115,50 - 156,00 | 0,532 |  |  |
| Lysine | Amino acids | 0 | 0% | X |  | 207,50 | 176,00 - 241,75 | 220,50 | 191,25 - 256,75 | 0,383 |  |  |
| Methionine | Amino acids | 0 | 0% | X |  | 19,40 | 16,38 - 22,58 | 20,30 | 18,45 - 23,60 | 0,151 |  |  |
| Ornithine | Amino acids | 0 | 0% | X |  | 64,65 | 55,53 - 82,35 | 78,40 | 58,05 - 110,00 | 0,086 |  |  |
| Phenylalanine | Amino acids | 0 | 0% | X |  | 58,85 | 51,78 - 64,73 | 59,35 | 52,43 - 65,63 | 0,471 |  |  |
| Proline | Amino acids | 0 | 0% | X |  | 189,00 | 141,75 - 220,25 | 190,00 | 153,50 - 260,00 | 0,398 |  |  |
| Serine | Amino acids | 0 | 0% | X |  | 110,50 | 92,58 - 133,00 | 108,50 | 88,70 - 132,75 | 0,656 |  |  |
| Threonine | Amino acids | 0 | 0% | X |  | 105,00 | 88,58 - 125,00 | 128,00 | 93,33 - 147,50 | 0,008 |  |  |
| Tryptophane | Amino acids | 0 | 0% | X |  | 54,85 | 47,55 - 67,43 | 59,70 | 51,85 - 64,53 | 0,313 |  |  |
| Tyrosine | Amino acids | 0 | 0% | X |  | 56,80 | 48,08 - 69,15 | 61,80 | 55,38 - 67,75 | 0,175 |  |  |
| Valine | Amino acids | 0 | 0% | X |  | 233,00 | 197,25 - 256,25 | 234,50 | 201,75 - 269,25 | 0,408 |  |  |
| Ac-Orn | Biogenic Amines | 51 | 71% | X |  |  |  |  |  |  |  |  |
| ADMA | Biogenic Amines | 10 | 14% | X |  | 0,53 | 0,41 - 0,82 | 0,67 | 0,57 - 0,93 | 0,056 |  |  |
| alpha-AAA | Biogenic Amines | 62 | 86% | X |  |  |  |  |  |  |  |  |
| c4-OH-Pro | Biogenic Amines | 72 | 100% | X |  |  |  |  |  |  |  |  |
| Carnosine | Biogenic Amines | 72 | 100% | X |  |  |  |  |  |  |  |  |
| Creatinine | Biogenic Amines | 0 | 0% | X |  | 65,55 | 55,33 - 75,08 | 66,50 | 59,20 - 81,30 | 0,464 |  |  |
| DOPA | Biogenic Amines | 72 | 100% | X |  |  |  |  |  |  |  |  |
| Dopamine | Biogenic Amines | 72 | 100% | X |  |  |  |  |  |  |  |  |
| Histamine | Biogenic Amines | 72 | 100% | X |  |  |  |  |  |  |  |  |
| Kynurenine | Biogenic Amines | 0 | 0% | X |  | 2,37 | 1,86 - 2,96 | 2,48 | 2,00 - 2,97 | 0,585 |  |  |
| Met-SO | Biogenic Amines | 72 | 100% | X |  |  |  |  |  |  |  |  |
| Nitro-Tyr | Biogenic Amines | 72 | 100% | X |  |  |  |  |  |  |  |  |
| PEA | Biogenic Amines | 72 | 100% | X |  |  |  |  |  |  |  |  |
| Putrescine | Biogenic Amines | 33 | 46% | X |  |  |  |  |  |  |  |  |
| Sarcosine | Biogenic Amines | 8 | 11% | X |  | 1,84 | 1,34 - 2,32 | 1,95 | 1,32 - 2,66 | 0,322 |  |  |
| SDMA | Biogenic Amines | 0 | 0% | X |  | 0,40 | 0,32 - 0,47 | 0,42 | 0,34 - 0,50 | 0,510 |  |  |
| Serotonin | Biogenic Amines | 29 | 40% | X |  |  |  |  |  |  |  |  |
| Spermidine | Biogenic Amines | 45 | 63% | X |  |  |  |  |  |  |  |  |
| Spermine | Biogenic Amines | 60 | 83% | X |  |  |  |  |  |  |  |  |
| t4-OH-Pro | Biogenic Amines | 40 | 56% | X |  |  |  |  |  |  |  |  |
| Taurine | Biogenic Amines | 29 | 40% | X |  |  |  |  |  |  |  |  |
| lysoPC a C14:0 | Glyercophospholipids | 72 | 100% |  | X |  |  |  |  |  |  |  |
| lysoPC a C16:0 | Glyercophospholipids | 0 | 0% |  | X | 52,30 | 41,65 - 64,75 | 56,15 | 41,83 - 67,95 | 0,454 |  |  |
| lysoPC a C16:1 | Glyercophospholipids | 0 | 0% |  | X | 1,58 | 1,25 - 2,03 | 1,70 | 1,35 - 1,97 | 0,371 |  |  |
| lysoPC a C17:0 | Glyercophospholipids | 0 | 0% |  | X | 0,98 | 0,69 - 1,08 | 0,91 | 0,67 - 1,20 | 0,727 |  |  |
| lysoPC a C18:0 | Glyercophospholipids | 0 | 0% |  | X | 13,30 | 10,23 - 17,43 | 14,65 | 11,48 - 18,78 | 0,319 |  |  |
| lysoPC a C18:1 | Glyercophospholipids | 0 | 0% |  | X | 11,15 | 9,75 - 14,45 | 12,50 | 8,61 - 14,90 | 0,853 |  |  |
| lysoPC a C18:2 | Glyercophospholipids | 0 | 0% |  | X | 13,80 | 10,70 - 17,33 | 13,80 | 9,28 - 18,30 | 0,817 |  |  |
| lysoPC a C20:3 | Glyercophospholipids | 0 | 0% |  | X | 1,24 | 0,95 - 1,68 | 1,22 | 1,01 - 1,68 | 0,796 |  |  |
| lysoPC a C20:4 | Glyercophospholipids | 0 | 0% |  | X | 3,97 | 3,36 - 5,26 | 3,63 | 2,62 - 4,46 | 0,083 |  |  |
| lysoPC a C24:0 | Glyercophospholipids | 1 | 1% |  | X | 0,07 | 0,06 - 0,08 | 0,07 | 0,07 - 0,08 | 0,689 |  |  |
| lysoPC a C26:0 | Glyercophospholipids | 25 | 35% |  | X | 0,08 | 0,07 - 0,10 | 0,09 | 0,07 - 0,11 | 0,327 |  |  |
| lysoPC a C26:1 | Glyercophospholipids | 28 | 39% |  | X | 0,05 | 0,04 - 0,06 | 0,05 | 0,03 - 0,06 | 0,791 |  |  |
| lysoPC a C28:0 | Glyercophospholipids | 18 | 25% |  | X | 0,11 | 0,10 - 0,12 | 0,12 | 0,11 - 0,14 | 0,044 |  |  |
| lysoPC a C28:1 | Glyercophospholipids | 0 | 0% |  | X | 0,19 | 0,16 - 0,21 | 0,18 | 0,14 - 0,23 | 0,408 |  |  |
| PC aa C24:0 | Glyercophospholipids | 39 | 54% |  | X |  |  |  |  |  |  |  |
| PC aa C26:0 | Glyercophospholipids | 71 | 99% |  | X |  |  |  |  |  |  |  |
| PC aa C28:1 | Glyercophospholipids | 0 | 0% |  | X | 2,22 | 1,95 - 2,59 | 2,32 | 1,83 - 2,65 | 0,892 |  |  |
| PC aa C30:0 | Glyercophospholipids | 0 | 0% |  | X | 3,46 | 2,62 - 3,74 | 3,30 | 2,44 - 4,55 | 0,796 |  |  |
| PC aa C30:2 | Glyercophospholipids | 36 | 50% |  | X |  |  |  |  |  |  |  |
| PC aa C32:0 | Glyercophospholipids | 0 | 0% |  | X | 14,65 | 12,85 - 16,10 | 13,45 | 12,10 - 16,85 | 0,411 |  |  |
| PC aa C32:1 | Glyercophospholipids | 0 | 0% |  | X | 17,75 | 13,13 - 27,20 | 16,75 | 13,60 - 28,40 | 0,848 |  |  |
| PC aa C32:2 | Glyercophospholipids | 0 | 0% |  | X | 3,74 | 2,79 - 4,92 | 3,78 | 2,94 - 4,69 | 0,857 |  |  |
| PC aa C32:3 | Glyercophospholipids | 0 | 0% |  | X | 0,43 | 0,37 - 0,50 | 0,41 | 0,33 - 0,53 | 0,499 |  |  |
| PC aa C34:1 | Glyercophospholipids | 0 | 0% |  | X | 233,50 | 215,00 - 284,50 | 225,00 | 195,00 - 289,25 | 0,669 |  |  |
| PC aa C34:2 | Glyercophospholipids | 0 | 0% |  | X | 401,00 | 360,00 - 433,50 | 386,00 | 349,00 - 436,00 | 0,440 |  |  |
| PC aa C34:3 | Glyercophospholipids | 0 | 0% |  | X | 14,85 | 11,90 - 18,65 | 14,00 | 11,85 - 21,73 | 0,800 |  |  |
| PC aa C34:4 | Glyercophospholipids | 0 | 0% |  | X | 1,94 | 1,33 - 2,38 | 1,74 | 1,25 - 2,44 | 0,933 |  |  |
| PC aa C36:0 | Glyercophospholipids | 0 | 0% |  | X | 1,82 | 1,49 - 2,41 | 2,03 | 1,54 - 2,59 | 0,401 |  |  |
| PC aa C36:1 | Glyercophospholipids | 0 | 0% |  | X | 40,50 | 32,45 - 47,23 | 40,85 | 33,90 - 52,33 | 0,506 |  |  |
| PC aa C36:2 | Glyercophospholipids | 0 | 0% |  | X | 204,00 | 179,25 - 219,75 | 199,50 | 172,50 - 244,00 | 0,600 |  |  |
| PC aa C36:3 | Glyercophospholipids | 0 | 0% |  | X | 119,00 | 101,75 - 142,50 | 113,50 | 100,13 - 142,50 | 0,566 |  |  |
| PC aa C36:4 | Glyercophospholipids | 0 | 0% |  | X | 186,50 | 155,50 - 230,50 | 179,00 | 132,00 - 209,75 | 0,295 |  |  |
| PC aa C36:5 | Glyercophospholipids | 0 | 0% |  | X | 23,05 | 16,35 - 31,40 | 25,30 | 15,30 - 32,03 | 0,955 |  |  |
| PC aa C36:6 | Glyercophospholipids | 0 | 0% |  | X | 0,81 | 0,57 - 1,01 | 0,78 | 0,58 - 1,11 | 0,562 |  |  |
| PC aa C38:0 | Glyercophospholipids | 0 | 0% |  | X | 1,79 | 1,51 - 2,29 | 1,92 | 1,54 - 2,33 | 0,569 |  |  |
| PC aa C38:1 | Glyercophospholipids | 0 | 0% |  | X | 0,81 | 0,61 - 1,04 | 0,74 | 0,64 - 0,95 | 0,660 |  |  |
| PC aa C38:3 | Glyercophospholipids | 0 | 0% |  | X | 40,25 | 31,03 - 47,58 | 38,60 | 32,63 - 46,60 | 0,866 |  |  |
| PC aa C38:4 | Glyercophospholipids | 0 | 0% |  | X | 89,55 | 73,53 - 108,25 | 87,90 | 64,48 - 96,83 | 0,267 |  |  |
| PC aa C38:5 | Glyercophospholipids | 0 | 0% |  | X | 45,60 | 36,05 - 55,48 | 41,20 | 32,68 - 52,18 | 0,241 |  |  |
| PC aa C38:6 | Glyercophospholipids | 0 | 0% |  | X | 62,80 | 50,60 - 71,93 | 57,85 | 43,33 - 82,50 | 0,644 |  |  |
| PC aa C40:1 | Glyercophospholipids | 53 | 74% |  | X |  |  |  |  |  |  |  |
| PC aa C40:2 | Glyercophospholipids | 0 | 0% |  | X | 0,20 | 0,15 - 0,28 | 0,20 | 0,17 - 0,28 | 0,748 |  |  |
| PC aa C40:3 | Glyercophospholipids | 0 | 0% |  | X | 0,39 | 0,33 - 0,47 | 0,41 | 0,34 - 0,46 | 0,892 |  |  |
| PC aa C40:4 | Glyercophospholipids | 0 | 0% |  | X | 2,27 | 1,93 - 2,78 | 2,24 | 2,07 - 2,68 | 0,919 |  |  |
| PC aa C40:5 | Glyercophospholipids | 0 | 0% |  | X | 6,55 | 5,43 - 7,64 | 5,87 | 5,04 - 7,57 | 0,499 |  |  |
| PC aa C40:6 | Glyercophospholipids | 0 | 0% |  | X | 17,70 | 13,58 - 22,30 | 17,85 | 12,60 - 25,35 | 0,870 |  |  |
| PC aa C42:0 | Glyercophospholipids | 0 | 0% |  | X | 0,32 | 0,25 - 0,42 | 0,33 | 0,24 - 0,37 | 0,593 |  |  |
| PC aa C42:1 | Glyercophospholipids | 0 | 0% |  | X | 0,15 | 0,12 - 0,18 | 0,16 | 0,12 - 0,18 | 0,973 |  |  |
| PC aa C42:2 | Glyercophospholipids | 2 | 3% |  | X | 0,14 | 0,12 - 0,18 | 0,14 | 0,11 - 0,17 | 0,796 |  |  |
| PC aa C42:4 | Glyercophospholipids | 0 | 0% |  | X | 0,12 | 0,09 - 0,15 | 0,11 | 0,09 - 0,13 | 0,604 |  |  |
| PC aa C42:5 | Glyercophospholipids | 0 | 0% |  | X | 0,21 | 0,19 - 0,26 | 0,22 | 0,19 - 0,27 | 0,719 |  |  |
| PC aa C42:6 | Glyercophospholipids | 0 | 0% |  | X | 0,26 | 0,22 - 0,29 | 0,27 | 0,20 - 0,32 | 0,787 |  |  |
| PC ae C30:0 | Glyercophospholipids | 0 | 0% |  | X | 0,28 | 0,23 - 0,32 | 0,29 | 0,21 - 0,40 | 0,371 |  |  |
| PC ae C30:1 | Glyercophospholipids | 9 | 13% |  | X | 0,07 | 0,04 - 0,10 | 0,07 | 0,03 - 0,11 | 0,822 |  |  |
| PC ae C30:2 | Glyercophospholipids | 0 | 0% |  | X | 0,07 | 0,06 - 0,08 | 0,07 | 0,06 - 0,08 | 0,681 |  |  |
| PC ae C32:1 | Glyercophospholipids | 0 | 0% |  | X | 2,56 | 2,28 - 2,93 | 2,56 | 2,20 - 3,21 | 0,955 |  |  |
| PC ae C32:2 | Glyercophospholipids | 0 | 0% |  | X | 0,66 | 0,54 - 0,76 | 0,66 | 0,57 - 0,80 | 0,636 |  |  |
| PC ae C34:0 | Glyercophospholipids | 0 | 0% |  | X | 1,49 | 1,21 - 1,65 | 1,38 | 1,06 - 1,82 | 0,731 |  |  |
| PC ae C34:1 | Glyercophospholipids | 0 | 0% |  | X | 10,00 | 8,99 - 11,70 | 9,67 | 7,91 - 11,60 | 0,566 |  |  |
| PC ae C34:2 | Glyercophospholipids | 0 | 0% |  | X | 10,05 | 8,80 - 11,48 | 10,55 | 8,83 - 13,45 | 0,421 |  |  |
| PC ae C34:3 | Glyercophospholipids | 0 | 0% |  | X | 5,95 | 4,71 - 7,69 | 6,63 | 5,27 - 7,89 | 0,547 |  |  |
| PC ae C36:0 | Glyercophospholipids | 0 | 0% |  | X | 0,75 | 0,63 - 0,87 | 0,78 | 0,64 - 0,90 | 0,973 |  |  |
| PC ae C36:1 | Glyercophospholipids | 0 | 0% |  | X | 8,10 | 6,20 - 9,43 | 7,39 | 5,93 - 9,75 | 0,562 |  |  |
| PC ae C36:2 | Glyercophospholipids | 0 | 0% |  | X | 12,45 | 10,65 - 15,18 | 11,45 | 9,96 - 15,28 | 0,660 |  |  |
| PC ae C36:3 | Glyercophospholipids | 0 | 0% |  | X | 6,24 | 5,43 - 7,00 | 6,68 | 5,66 - 7,93 | 0,213 |  |  |
| PC ae C36:4 | Glyercophospholipids | 0 | 0% |  | X | 15,60 | 13,05 - 20,00 | 16,40 | 13,08 - 19,88 | 0,875 |  |  |
| PC ae C36:5 | Glyercophospholipids | 0 | 0% |  | X | 10,70 | 9,36 - 13,95 | 10,75 | 8,51 - 13,55 | 0,558 |  |  |
| PC ae C38:0 | Glyercophospholipids | 0 | 0% |  | X | 1,45 | 1,24 - 1,96 | 1,63 | 1,26 - 2,02 | 0,660 |  |  |
| PC ae C38:1 | Glyercophospholipids | 0 | 0% |  | X | 0,41 | 0,30 - 0,90 | 0,48 | 0,41 - 0,86 | 0,188 |  |  |
| PC ae C38:2 | Glyercophospholipids | 0 | 0% |  | X | 1,59 | 1,17 - 2,09 | 1,62 | 1,28 - 2,32 | 0,765 |  |  |
| PC ae C38:3 | Glyercophospholipids | 0 | 0% |  | X | 3,92 | 3,23 - 5,28 | 3,91 | 3,24 - 5,21 | 0,761 |  |  |
| PC ae C38:4 | Glyercophospholipids | 0 | 0% |  | X | 11,30 | 9,48 - 13,08 | 10,70 | 9,10 - 12,30 | 0,424 |  |  |
| PC ae C38:5 | Glyercophospholipids | 0 | 0% |  | X | 14,20 | 12,03 - 18,23 | 14,50 | 12,58 - 17,73 | 0,740 |  |  |
| PC ae C38:6 | Glyercophospholipids | 0 | 0% |  | X | 5,45 | 4,57 - 7,13 | 5,89 | 4,58 - 6,98 | 0,978 |  |  |
| PC ae C40:1 | Glyercophospholipids | 0 | 0% |  | X | 0,92 | 0,75 - 1,11 | 0,84 | 0,76 - 1,03 | 0,386 |  |  |
| PC ae C40:2 | Glyercophospholipids | 0 | 0% |  | X | 1,48 | 1,17 - 1,97 | 1,46 | 1,21 - 1,66 | 0,550 |  |  |
| PC ae C40:3 | Glyercophospholipids | 0 | 0% |  | X | 0,85 | 0,70 - 1,33 | 0,97 | 0,70 - 1,30 | 0,951 |  |  |
| PC ae C40:4 | Glyercophospholipids | 0 | 0% |  | X | 1,89 | 1,61 - 2,44 | 1,89 | 1,56 - 2,28 | 0,765 |  |  |
| PC ae C40:5 | Glyercophospholipids | 0 | 0% |  | X | 2,95 | 2,36 - 3,83 | 2,80 | 2,27 - 3,36 | 0,395 |  |  |
| PC ae C40:6 | Glyercophospholipids | 0 | 0% |  | X | 3,00 | 2,53 - 3,60 | 2,96 | 2,52 - 3,54 | 0,624 |  |  |
| PC ae C42:0 | Glyercophospholipids | 71 | 99% |  | X |  |  |  |  |  |  |  |
| PC ae C42:1 | Glyercophospholipids | 2 | 3% |  | X | 0,26 | 0,22 - 0,34 | 0,26 | 0,21 - 0,30 | 0,636 |  |  |
| PC ae C42:2 | Glyercophospholipids | 0 | 0% |  | X | 0,41 | 0,33 - 0,48 | 0,38 | 0,31 - 0,47 | 0,804 |  |  |
| PC ae C42:3 | Glyercophospholipids | 0 | 0% |  | X | 0,47 | 0,39 - 0,61 | 0,48 | 0,37 - 0,59 | 0,901 |  |  |
| PC ae C42:4 | Glyercophospholipids | 0 | 0% |  | X | 0,59 | 0,50 - 0,71 | 0,60 | 0,45 - 0,69 | 0,748 |  |  |
| PC ae C42:5 | Glyercophospholipids | 0 | 0% |  | X | 1,53 | 1,32 - 1,97 | 1,55 | 1,34 - 1,84 | 0,551 |  |  |
| PC ae C44:3 | Glyercophospholipids | 34 | 47% |  | X |  |  |  |  |  |  |  |
| PC ae C44:4 | Glyercophospholipids | 0 | 0% |  | X | 0,23 | 0,19 - 0,28 | 0,25 | 0,19 - 0,29 | 0,389 |  |  |
| PC ae C44:5 | Glyercophospholipids | 0 | 0% |  | X | 1,17 | 0,87 - 1,33 | 1,03 | 0,86 - 1,24 | 0,220 |  |  |
| PC ae C44:6 | Glyercophospholipids | 0 | 0% |  | X | 0,68 | 0,50 - 0,81 | 0,60 | 0,51 - 0,76 | 0,303 |  |  |
| SM (OH) C14:1 | Sphingolipids | 0 | 0% |  | X | 4,63 | 3,76 - 5,16 | 4,00 | 3,30 - 5,15 | 0,237 |  |  |
| SM (OH) C16:1 | Sphingolipids | 0 | 0% |  | X | 2,21 | 1,88 - 2,71 | 2,02 | 1,59 - 2,47 | 0,224 |  |  |
| SM (OH) C22:1 | Sphingolipids | 0 | 0% |  | X | 6,63 | 5,24 - 8,03 | 6,35 | 4,79 - 7,15 | 0,207 |  |  |
| SM (OH) C22:2 | Sphingolipids | 0 | 0% |  | X | 5,55 | 4,65 - 6,76 | 5,31 | 3,97 - 6,23 | 0,146 |  |  |
| SM (OH) C24:1 | Sphingolipids | 0 | 0% |  | X | 0,52 | 0,45 - 0,69 | 0,56 | 0,41 - 0,65 | 0,875 |  |  |
| SM C16:0 | Sphingolipids | 0 | 0% |  | X | 78,50 | 65,85 - 89,35 | 75,60 | 61,40.84,03 | 0,258 |  |  |
| SM C16:1 | Sphingolipids | 0 | 0% |  | X | 10,80 | 9,18 - 11,88 | 9,94 | 8,76 - 12,10 | 0,562 |  |  |
| SM C18:0 | Sphingolipids | 0 | 0% |  | X | 15,65 | 13,43 - 19,35 | 15,00 | 13,10 - 17,38 | 0,330 |  |  |
| SM C18:1 | Sphingolipids | 0 | 0% |  | X | 7,43 | 6,00 - 8,38 | 6,92 | 5,40 - 7,66 | 0,215 |  |  |
| SM C20:2 | Sphingolipids | 0 | 0% |  | X | 0,22 | 0,17 - 0,27 | 0,20 | 0,15 - 0,27 | 0,517 |  |  |
| SM C22:3 | Sphingolipids | 70 | 97% |  | X |  |  |  |  |  |  |  |
| SM C24:0 | Sphingolipids | 0 | 0% |  | X | 10,20 | 8,64 - 12,15 | 10,30 | 8,13 - 12,35 | 0,620 |  |  |
| SM C24:1 | Sphingolipids | 0 | 0% |  | X | 24,15 | 21,75 - 29,18 | 25,35 | 19,23 - 30,55 | 0,562 |  |  |
| SM C26:0 | Sphingolipids | 0 | 0% |  | X | 0,07 | 0,05 - 0,09 | 0,07 | 0,05 - 0,09 | 0,946 |  |  |
| SM C26:1 | Sphingolipids | 0 | 0% |  | X | 0,18 | 0,15 - 0,24 | 0,16 | 0,12 - 0,23 | 0,398 |  |  |
| H1 | Sum of hexoses | 0 | 0% |  | X | 4844,00 | 4325,50 - 5364,50 | 4215,50 | 3791,00 - 5086,00 | 0,018 |  |  |
|  |  |  |  |  |  |  |  |  |  | | |  |
|  | Exclusion of the metabolite from the statistical evaluation | | | | | |  |  |  | | |  |

**Metabolic profile of patients with PPGL vs controls.**


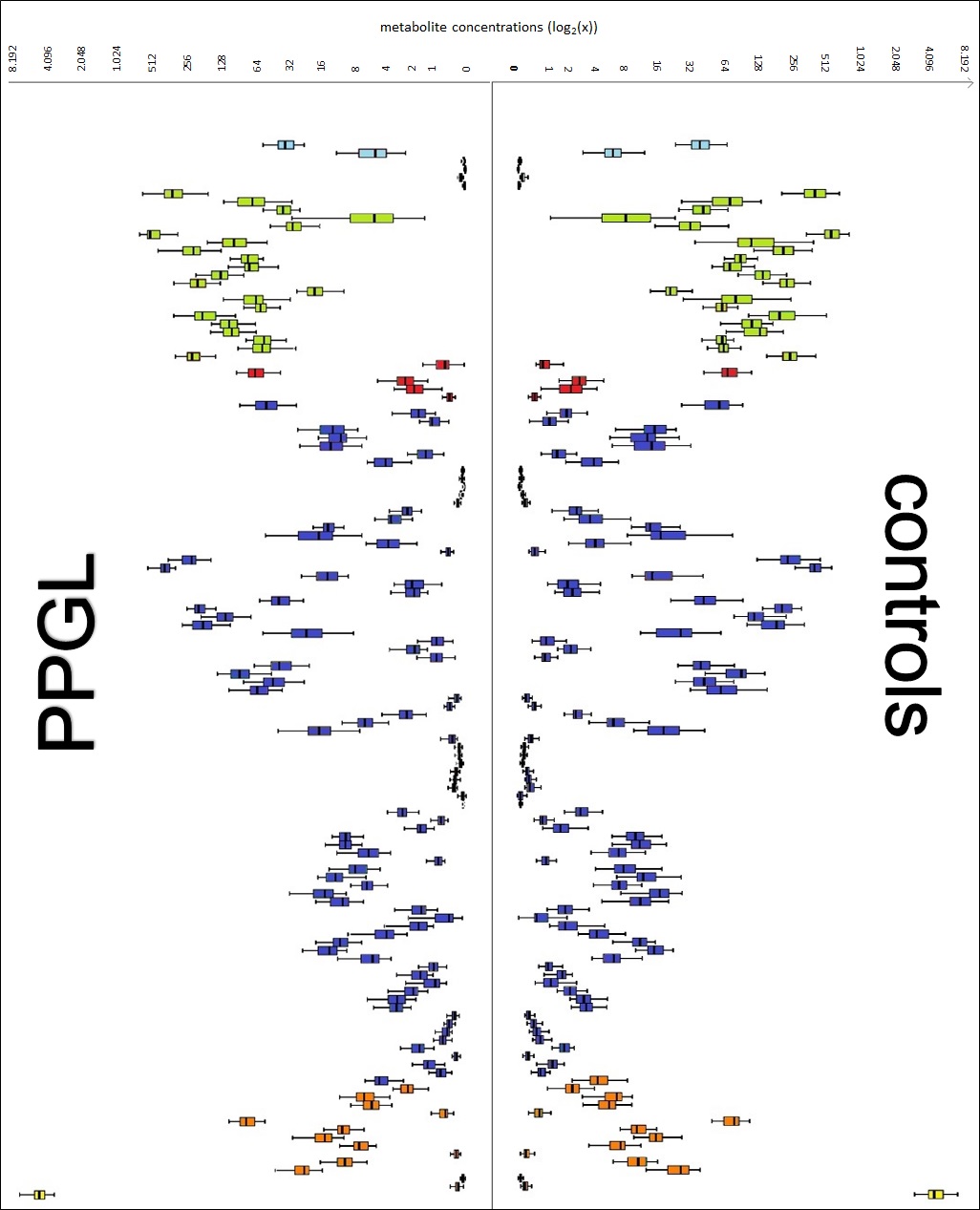


Log transformed quantitative date (mediam [µmol/l], min/ max, IQR) of 130 metabolites in 6 groups of metabolites are shown. Acylcarnitines (light blue), amino acids (green), biogenic amines (red), glycerophospholipids (blue), sphingolipids (orange), sum of hexose (yellow).

**Correlations PPGL vs controls**

|  | | Plasma NMN | Plasma MN | Plasma MTY | Urinary free NE | Urinary free EPI | Urinary free DA | TUC | Histidine | Threonine | lysoPC a C28:0 | Sumo f hexoses |
| --- | --- | --- | --- | --- | --- | --- | --- | --- | --- | --- | --- | --- |
| Plasma NMN | r_s_ | 1,000 | ,439** | ,703** | ,628** | ,281* | -0,009 | ,382** | -,287* | -0,229 | -0,169 | ,337** |
|  | p-Wert |  | 0,000 | 0,000 | 0,000 | 0,028 | 0,947 | 0,003 | 0,015 | 0,053 | 0,157 | 0,004 |
| Plasma MN | r_s_ | ,439** | 1,000 | ,405** | 0,161 | ,593** | 0,045 | 0,145 | -0,219 | -0,054 | -0,147 | ,276* |
|  | p-Wert | 0,000 |  | 0,001 | 0,211 | 0,000 | 0,733 | 0,277 | 0,065 | 0,655 | 0,217 | 0,019 |
| Plasma MTY | r_s_ | ,703** | ,405** | 1,000 | ,386** | 0,187 | 0,010 | ,322* | -,242* | -,266* | -0,144 | ,339** |
|  | p-Wert | 0,000 | 0,001 |  | 0,002 | 0,156 | 0,943 | 0,015 | 0,044 | 0,026 | 0,233 | 0,004 |
| Urinary free NE | r_s_ | ,628** | 0,161 | ,386** | 1,000 | ,448** | ,287* | ,650** | -0,239 | -,255* | -0,212 | ,437** |
|  | p-Wert | 0,000 | 0,211 | 0,002 |  | 0,000 | 0,027 | 0,000 | 0,061 | 0,045 | 0,097 | 0,000 |
| Urinary free EPI | r_s_ | ,281* | ,593** | 0,187 | ,448** | 1,000 | ,457** | ,566** | -,408** | -,304* | -0,219 | 0,145 |
|  | p-Wert | 0,028 | 0,000 | 0,156 | 0,000 |  | 0,000 | 0,000 | 0,001 | 0,017 | 0,090 | 0,264 |
| Urinary free DA | r_s_ | -0,009 | 0,045 | 0,010 | ,287* | ,457** | 1,000 | ,833** | -,300* | -0,161 | -,260* | -0,046 |
|  | p-Wert | 0,947 | 0,733 | 0,943 | 0,027 | 0,000 |  | 0,000 | 0,021 | 0,224 | 0,047 | 0,728 |
| TUC | r_s_ | ,382** | 0,145 | ,322* | ,650** | ,566** | ,833** | 1,000 | -,407** | -,275* | -,269* | 0,221 |
|  | p-Wert | 0,003 | 0,277 | 0,015 | 0,000 | 0,000 | 0,000 |  | 0,002 | 0,037 | 0,041 | 0,096 |
| Histidine | r_s_ | -,287* | -0,219 | -,242* | -0,239 | -,408** | -,300* | -,407** | 1,000 | ,370** | ,270* | -0,091 |
|  | p-Wert | 0,015 | 0,065 | 0,044 | 0,061 | 0,001 | 0,021 | 0,002 |  | 0,001 | 0,022 | 0,449 |
| Threonine | r_s_ | -0,229 | -0,054 | -,266* | -,255* | -,304* | -0,161 | -,275* | ,370** | 1,000 | 0,129 | -0,112 |
|  | p-Wert | 0,053 | 0,655 | 0,026 | 0,045 | 0,017 | 0,224 | 0,037 | 0,001 |  | 0,278 | 0,350 |
| lysoPC a C28:0 | r_s_ | -0,169 | -0,147 | -0,144 | -0,212 | -0,219 | -,260* | -,269* | ,270* | 0,129 | 1,000 | -0,211 |
|  | p-Wert | 0,157 | 0,217 | 0,233 | 0,097 | 0,090 | 0,047 | 0,041 | 0,022 | 0,278 |  | 0,075 |
| Sum of hexoses | r_s_ | ,337** | ,276* | ,339** | ,437** | 0,145 | -0,046 | 0,221 | -0,091 | -0,112 | -0,211 | 1,000 |
|  | p-Wert | 0,004 | 0,019 | 0,004 | 0,000 | 0,264 | 0,728 | 0,096 | 0,449 | 0,350 | 0,075 |  |

* The correlation is significant at level 0.05 (two-sided). ** The correlation is significant at level 0.01 (two-sided). TUC, total urine catecholamines.

**Correlations female PPGL vs control**

|  | | Plasma NMN | Plasma MN | Plasma MTY | Urinary free NE | Urinary free NE | Urinary free NE | Urinary free NE | PC ae C38:1 | PC aa C38:4 | PC aa C36:4 | lysoPC a C20:4 | Histidine |
| --- | --- | --- | --- | --- | --- | --- | --- | --- | --- | --- | --- | --- | --- |
| Plasma NMN | r_s_ | 1,000 | ,555^**^ | ,765^**^ | ,672^**^ | ,405^*^ | -0,007 | 0,345 | -,368^*^ | 0,325 | ,356^*^ | ,471^**^ | -,428^*^ |
|  | p-Wert |  | 0,001 | 0,000 | 0,000 | 0,036 | 0,971 | 0,078 | 0,032 | 0,060 | 0,039 | 0,005 | 0,012 |
| Plasma MN | r_s_ | ,555^**^ | 1,000 | ,563^**^ | 0,234 | ,619^**^ | 0,097 | 0,246 | -,474^**^ | ,535^**^ | ,514^**^ | 0,273 | -0,339 |
|  | p-Wert | 0,001 |  | 0,001 | 0,240 | 0,001 | 0,630 | 0,216 | 0,005 | 0,001 | 0,002 | 0,118 | 0,050 |
| Plasma MTY | r_s_ | ,765^**^ | ,563^**^ | 1,000 | ,512^**^ | ,405^*^ | 0,048 | 0,366 | -0,137 | ,508^**^ | ,525^**^ | ,438^*^ | -0,301 |
|  | p-Wert | 0,000 | 0,001 |  | 0,009 | 0,044 | 0,821 | 0,072 | 0,453 | 0,003 | 0,002 | 0,012 | 0,095 |
| Urinary free NE | r_s_ | ,672^**^ | 0,234 | ,512^**^ | 1,000 | ,533^**^ | 0,196 | ,582^**^ | -0,353 | 0,120 | 0,117 | ,537^**^ | -0,364 |
|  | p-Wert | 0,000 | 0,240 | 0,009 |  | 0,004 | 0,327 | 0,001 | 0,071 | 0,550 | 0,560 | 0,004 | 0,062 |
| Urinary free EPI | r_s_ | ,405^*^ | ,619^**^ | ,405^*^ | ,533^**^ | 1,000 | ,474^*^ | ,606^**^ | -,531^**^ | 0,197 | 0,022 | ,412^*^ | -,434^*^ |
|  | p-Wert | 0,036 | 0,001 | 0,044 | 0,004 |  | 0,013 | 0,001 | 0,004 | 0,325 | 0,913 | 0,033 | 0,024 |
| Urinary free DA | r_s_ | -0,007 | 0,097 | 0,048 | 0,196 | ,474^*^ | 1,000 | ,838^**^ | -0,048 | -0,093 | -0,256 | 0,302 | -0,294 |
|  | p-Wert | 0,971 | 0,630 | 0,821 | 0,327 | 0,013 |  | 0,000 | 0,812 | 0,643 | 0,197 | 0,126 | 0,137 |
| TUC | r_s_ | 0,345 | 0,246 | 0,366 | ,582^**^ | ,606^**^ | ,838^**^ | 1,000 | -0,225 | 0,002 | -0,123 | ,538^**^ | -,479^*^ |
|  | p-Wert | 0,078 | 0,216 | 0,072 | 0,001 | 0,001 | 0,000 |  | 0,260 | 0,993 | 0,540 | 0,004 | 0,012 |
| PC ae C38:1 | r_s_ | -,368^*^ | -,474^**^ | -0,137 | -0,353 | -,531^**^ | -0,048 | -0,225 | 1,000 | -0,298 | -0,150 | -,354^*^ | 0,230 |
|  | p-Wert | 0,032 | 0,005 | 0,453 | 0,071 | 0,004 | 0,812 | 0,260 |  | 0,087 | 0,397 | 0,040 | 0,190 |
| PC aa C38:4 | r_s_ | 0,325 | ,535^**^ | ,508^**^ | 0,120 | 0,197 | -0,093 | 0,002 | -0,298 | 1,000 | ,856^**^ | ,442^**^ | -0,065 |
|  | p-Wert | 0,060 | 0,001 | 0,003 | 0,550 | 0,325 | 0,643 | 0,993 | 0,087 |  | 0,000 | 0,009 | 0,717 |
| PC aa C36:4 | r_s_ | ,356^*^ | ,514^**^ | ,525^**^ | 0,117 | 0,022 | -0,256 | -0,123 | -0,150 | ,856^**^ | 1,000 | 0,252 | 0,023 |
|  | p-Wert | 0,039 | 0,002 | 0,002 | 0,560 | 0,913 | 0,197 | 0,540 | 0,397 | 0,000 |  | 0,150 | 0,896 |
| lysoPC a C20:4 | r_s_ | ,471^**^ | 0,273 | ,438^*^ | ,537^**^ | ,412^*^ | 0,302 | ,538^**^ | -,354^*^ | ,442^**^ | 0,252 | 1,000 | -0,180 |
|  | p-Wert | 0,005 | 0,118 | 0,012 | 0,004 | 0,033 | 0,126 | 0,004 | 0,040 | 0,009 | 0,150 |  | 0,309 |
| Histidine | r_s_ | -,428^*^ | -0,339 | -0,301 | -0,364 | -,434^*^ | -0,294 | -,479^*^ | 0,230 | -0,065 | 0,023 | -0,180 | 1,000 |
|  | p-Wert | 0,012 | 0,050 | 0,095 | 0,062 | 0,024 | 0,137 | 0,012 | 0,190 | 0,717 | 0,896 | 0,309 |  |

* The correlation is significant at level 0.05 (two-sided). ** The correlation is significant at level 0.01 (two-sided). TUC, total urine catecholamines.

**Correlations male PPGL vs control**

|  | | Plasma NMN | Plasma MN | Plasma MTY | Urinary free NE | Urinary free NE | Urinary free NE | Urinary free NE | Threonine | lysoPC a C16:1 | PC ae C30:2 | SM (OH) C14:1 | Sumo f hexoses |
| --- | --- | --- | --- | --- | --- | --- | --- | --- | --- | --- | --- | --- | --- |
| Plasma NMN | r_s_ | 1,000 | 0,213 | ,633** | ,586** | 0,090 | 0,000 | ,385* | -,328* | -0,295 | 0,244 | 0,266 | ,381* |
|  | p-Wert |  | 0,200 | 0,000 | 0,000 | 0,612 | 1,000 | 0,032 | 0,044 | 0,072 | 0,140 | 0,107 | 0,018 |
| Plasma MN | r_s_ | 0,213 | 1,000 | 0,212 | 0,081 | ,580** | 0,003 | -0,012 | -0,206 | 0,163 | -0,168 | -0,157 | 0,068 |
|  | p-Wert | 0,200 |  | 0,201 | 0,642 | 0,000 | 0,988 | 0,948 | 0,215 | 0,329 | 0,314 | 0,347 | 0,686 |
| Plasma MTY | r_s_ | ,633** | 0,212 | 1,000 | 0,316 | -0,027 | -0,038 | 0,244 | -0,226 | -0,147 | 0,071 | -0,060 | 0,306 |
|  | p-Wert | 0,000 | 0,201 |  | 0,064 | 0,880 | 0,837 | 0,186 | 0,173 | 0,380 | 0,672 | 0,720 | 0,062 |
| Urinary free NE | r_s_ | ,586** | 0,081 | 0,316 | 1,000 | ,361* | ,411* | ,722** | -0,269 | -0,329 | 0,185 | 0,071 | ,425* |
|  | p-Wert | 0,000 | 0,642 | 0,064 |  | 0,036 | 0,019 | 0,000 | 0,118 | 0,053 | 0,287 | 0,685 | 0,011 |
| Urinary free EPI | r_s_ | 0,090 | ,580** | -0,027 | ,361* | 1,000 | ,446* | ,499** | -,345* | 0,042 | -0,181 | -0,151 | -0,020 |
|  | p-Wert | 0,612 | 0,000 | 0,880 | 0,036 |  | 0,012 | 0,004 | 0,046 | 0,813 | 0,306 | 0,393 | 0,911 |
| Urinary free DA | r_s_ | 0,000 | 0,003 | -0,038 | ,411* | ,446* | 1,000 | ,845** | -0,085 | -0,102 | -0,208 | -0,253 | -0,097 |
|  | p-Wert | 1,000 | 0,988 | 0,837 | 0,019 | 0,012 |  | 0,000 | 0,643 | 0,579 | 0,254 | 0,162 | 0,598 |
| TUC | r_s_ | ,385* | -0,012 | 0,244 | ,722** | ,499** | ,845** | 1,000 | -0,212 | -0,184 | -0,040 | -0,084 | 0,206 |
|  | p-Wert | 0,032 | 0,948 | 0,186 | 0,000 | 0,004 | 0,000 |  | 0,252 | 0,322 | 0,832 | 0,654 | 0,265 |
| Threonine | r_s_ | -,328* | -0,206 | -0,226 | -0,269 | -,345* | -0,085 | -0,212 | 1,000 | 0,051 | -,414** | -,428** | 0,004 |
|  | p-Wert | 0,044 | 0,215 | 0,173 | 0,118 | 0,046 | 0,643 | 0,252 |  | 0,761 | 0,010 | 0,007 | 0,979 |
| lysoPC a C16:1 | r_s_ | -0,295 | 0,163 | -0,147 | -0,329 | 0,042 | -0,102 | -0,184 | 0,051 | 1,000 | -0,239 | -0,283 | -0,097 |
|  | p-Wert | 0,072 | 0,329 | 0,380 | 0,053 | 0,813 | 0,579 | 0,322 | 0,761 |  | 0,149 | 0,085 | 0,563 |
| PC ae C30:2 | r_s_ | 0,244 | -0,168 | 0,071 | 0,185 | -0,181 | -0,208 | -0,040 | -,414** | -0,239 | 1,000 | ,845** | 0,120 |
|  | p-Wert | 0,140 | 0,314 | 0,672 | 0,287 | 0,306 | 0,254 | 0,832 | 0,010 | 0,149 |  | 0,000 | 0,475 |
| SM (OH) C14:1 | r_s_ | 0,266 | -0,157 | -0,060 | 0,071 | -0,151 | -0,253 | -0,084 | -,428** | -0,283 | ,845** | 1,000 | 0,070 |
|  | p-Wert | 0,107 | 0,347 | 0,720 | 0,685 | 0,393 | 0,162 | 0,654 | 0,007 | 0,085 | 0,000 |  | 0,677 |
| Sum of hexoses | r_s_ | ,381* | 0,068 | 0,306 | ,425* | -0,020 | -0,097 | 0,206 | 0,004 | -0,097 | 0,120 | 0,070 | 1,000 |
|  | p-Wert | 0,018 | 0,686 | 0,062 | 0,011 | 0,911 | 0,598 | 0,265 | 0,979 | 0,563 | 0,475 | 0,677 |  |

* The correlation is significant at level 0.05 (two-sided). ** The correlation is significant at level 0.01 (two-sided). TUC, total urine catecholamines.

**Correlations adrenergic phenotype PPGL vs control**

|  | | Plasma NMN | Plasma MN | Plasma MTY | Urinary free NE | Urinary free NE | Urinary free NE | Urinary free NE | Glycin | Histidine | lysoPC a C20:4 | lysoPC a C28:0 |
| --- | --- | --- | --- | --- | --- | --- | --- | --- | --- | --- | --- | --- |
| Plasma NMN | r_s_ | 1,000 | ,862** | ,684** | ,521** | ,703** | -0,061 | 0,279 | ,464** | -,597** | 0,353 | -,371* |
|  | p-Wert |  | 0,000 | 0,000 | 0,005 | 0,000 | 0,769 | 0,168 | 0,010 | 0,000 | 0,056 | 0,043 |
| Plasma MN | r_s_ | ,862** | 1,000 | ,675** | 0,304 | ,762** | -0,088 | 0,199 | ,490** | -,494** | 0,317 | -0,236 |
|  | p-Wert | 0,000 |  | 0,000 | 0,123 | 0,000 | 0,670 | 0,329 | 0,006 | 0,006 | 0,088 | 0,210 |
| Plasma MTY | r_s_ | ,684** | ,675** | 1,000 | 0,212 | ,497* | -0,147 | 0,117 | 0,258 | -,535** | 0,082 | -0,336 |
|  | p-Wert | 0,000 | 0,000 |  | 0,310 | 0,012 | 0,492 | 0,585 | 0,186 | 0,003 | 0,679 | 0,080 |
| Urinary free NE | r_s_ | ,521** | 0,304 | 0,212 | 1,000 | ,676** | 0,357 | ,670** | 0,280 | -,412* | ,461* | -0,345 |
|  | p-Wert | 0,005 | 0,123 | 0,310 |  | 0,000 | 0,074 | 0,000 | 0,157 | 0,033 | 0,016 | 0,078 |
| Urinary free EPI | r_s_ | ,703** | ,762** | ,497* | ,676** | 1,000 | 0,160 | ,488* | 0,376 | -,499** | ,477* | -0,327 |
|  | p-Wert | 0,000 | 0,000 | 0,012 | 0,000 |  | 0,434 | 0,011 | 0,053 | 0,008 | 0,012 | 0,096 |
| Urinary free DA | r_s_ | -0,061 | -0,088 | -0,147 | 0,357 | 0,160 | 1,000 | ,883** | 0,108 | -0,315 | 0,087 | -0,211 |
|  | p-Wert | 0,769 | 0,670 | 0,492 | 0,074 | 0,434 |  | 0,000 | 0,600 | 0,117 | 0,674 | 0,301 |
| TUC | r_s_ | 0,279 | 0,199 | 0,117 | ,670** | ,488* | ,883** | 1,000 | 0,297 | -,459* | 0,283 | -0,297 |
|  | p-Wert | 0,168 | 0,329 | 0,585 | 0,000 | 0,011 | 0,000 |  | 0,141 | 0,018 | 0,161 | 0,141 |
| Glycin | r_s_ | ,464** | ,490** | 0,258 | 0,280 | 0,376 | 0,108 | 0,297 | 1,000 | -0,087 | ,401* | 0,020 |
|  | p-Wert | 0,010 | 0,006 | 0,186 | 0,157 | 0,053 | 0,600 | 0,141 |  | 0,646 | 0,028 | 0,916 |
| Histidine | r_s_ | -,597** | -,494** | -,535** | -,412* | -,499** | -0,315 | -,459* | -0,087 | 1,000 | -0,052 | 0,337 |
|  | p-Wert | 0,000 | 0,006 | 0,003 | 0,033 | 0,008 | 0,117 | 0,018 | 0,646 |  | 0,785 | 0,069 |
| lysoPC a C20:4 | r_s_ | 0,353 | 0,317 | 0,082 | ,461* | ,477* | 0,087 | 0,283 | ,401* | -0,052 | 1,000 | -0,218 |
|  | p-Wert | 0,056 | 0,088 | 0,679 | 0,016 | 0,012 | 0,674 | 0,161 | 0,028 | 0,785 |  | 0,247 |
| lysoPC a C28:0 | r_s_ | -,371* | -0,236 | -0,336 | -0,345 | -0,327 | -0,211 | -0,297 | 0,020 | 0,337 | -0,218 | 1,000 |
|  | p-Wert | 0,043 | 0,210 | 0,080 | 0,078 | 0,096 | 0,301 | 0,141 | 0,916 | 0,069 | 0,247 |  |

* The correlation is significant at level 0.05 (two-sided). ** The correlation is significant at level 0.01 (two-sided). TUC, total urine catecholamines.

**Correlations noradrenergic PPGL vs control**

|  | | Plasma NMN | Plasma MN | Plasma MTY | Urinary free NE | Urinary free NE | Urinary free NE | Urinary free NE | C0 | Asparagine | Threonine | ADMA |
| --- | --- | --- | --- | --- | --- | --- | --- | --- | --- | --- | --- | --- |
| Plasma NMN | r_s_ | 1,000 | ,307* | ,684** | ,676** | 0,056 | 0,035 | ,430* | -0,226 | -0,252 | -,344* | -0,258 |
|  | p-Wert |  | 0,048 | 0,000 | 0,000 | 0,751 | 0,848 | 0,014 | 0,149 | 0,108 | 0,026 | 0,099 |
| Plasma MN | r_s_ | ,307* | 1,000 | ,408** | 0,215 | 0,309 | 0,122 | 0,146 | 0,201 | 0,129 | 0,064 | -0,041 |
|  | p-Wert | 0,048 |  | 0,007 | 0,215 | 0,076 | 0,499 | 0,424 | 0,202 | 0,416 | 0,687 | 0,795 |
| Plasma MTY | r_s_ | ,684** | ,408** | 1,000 | ,454** | 0,038 | 0,082 | ,390* | -0,234 | -0,124 | -,351* | -0,132 |
|  | p-Wert | 0,000 | 0,007 |  | 0,006 | 0,831 | 0,650 | 0,027 | 0,136 | 0,435 | 0,023 | 0,404 |
| Urinary free NE | r_s_ | ,676** | 0,215 | ,454** | 1,000 | ,466** | 0,276 | ,643** | -0,114 | -0,254 | -,472** | -,399* |
|  | p-Wert | 0,000 | 0,215 | 0,006 |  | 0,006 | 0,120 | 0,000 | 0,515 | 0,141 | 0,004 | 0,018 |
| Urinary free EPI | r_s_ | 0,056 | 0,309 | 0,038 | ,466** | 1,000 | ,698** | ,674** | -0,089 | -0,060 | -0,199 | -0,029 |
|  | p-Wert | 0,751 | 0,076 | 0,831 | 0,006 |  | 0,000 | 0,000 | 0,616 | 0,736 | 0,260 | 0,871 |
| Urinary free DA | r_s_ | 0,035 | 0,122 | 0,082 | 0,276 | ,698** | 1,000 | ,829** | -0,342 | -0,116 | -0,307 | -0,001 |
|  | p-Wert | 0,848 | 0,499 | 0,650 | 0,120 | 0,000 |  | 0,000 | 0,051 | 0,522 | 0,082 | 0,997 |
| TUC | r_s_ | ,430* | 0,146 | ,390* | ,643** | ,674** | ,829** | 1,000 | -0,290 | -0,193 | -,494** | -0,195 |
|  | p-Wert | 0,014 | 0,424 | 0,027 | 0,000 | 0,000 | 0,000 |  | 0,108 | 0,289 | 0,004 | 0,285 |
| C0 | r_s_ | -0,226 | 0,201 | -0,234 | -0,114 | -0,089 | -0,342 | -0,290 | 1,000 | ,452** | 0,240 | 0,114 |
|  | p-Wert | 0,149 | 0,202 | 0,136 | 0,515 | 0,616 | 0,051 | 0,108 |  | 0,003 | 0,125 | 0,472 |
| Asparagine | r_s_ | -0,252 | 0,129 | -0,124 | -0,254 | -0,060 | -0,116 | -0,193 | ,452** | 1,000 | ,534** | -0,018 |
|  | p-Wert | 0,108 | 0,416 | 0,435 | 0,141 | 0,736 | 0,522 | 0,289 | 0,003 |  | 0,000 | 0,912 |
| Threonine | r_s_ | -,344* | 0,064 | -,351* | -,472** | -0,199 | -0,307 | -,494** | 0,240 | ,534** | 1,000 | 0,152 |
|  | p-Wert | 0,026 | 0,687 | 0,023 | 0,004 | 0,260 | 0,082 | 0,004 | 0,125 | 0,000 |  | 0,338 |
| ADMA | r_s_ | -0,258 | -0,041 | -0,132 | -,399* | -0,029 | -0,001 | -0,195 | 0,114 | -0,018 | 0,152 | 1,000 |
|  | p-Wert | 0,099 | 0,795 | 0,404 | 0,018 | 0,871 | 0,997 | 0,285 | 0,472 | 0,912 | 0,338 |  |

* The correlation is significant at level 0.05 (two-sided). ** The correlation is significant at level 0.01 (two-sided). TUC, total urine catecholamines.

**Model evaluation in the test dataset. The models were tested to predict ‘Class’ on the 20% testing split, which generates a confusion matrix for the predicted vs actual values. ROC curves compare the AUC and true positive rate of the 3 models.**


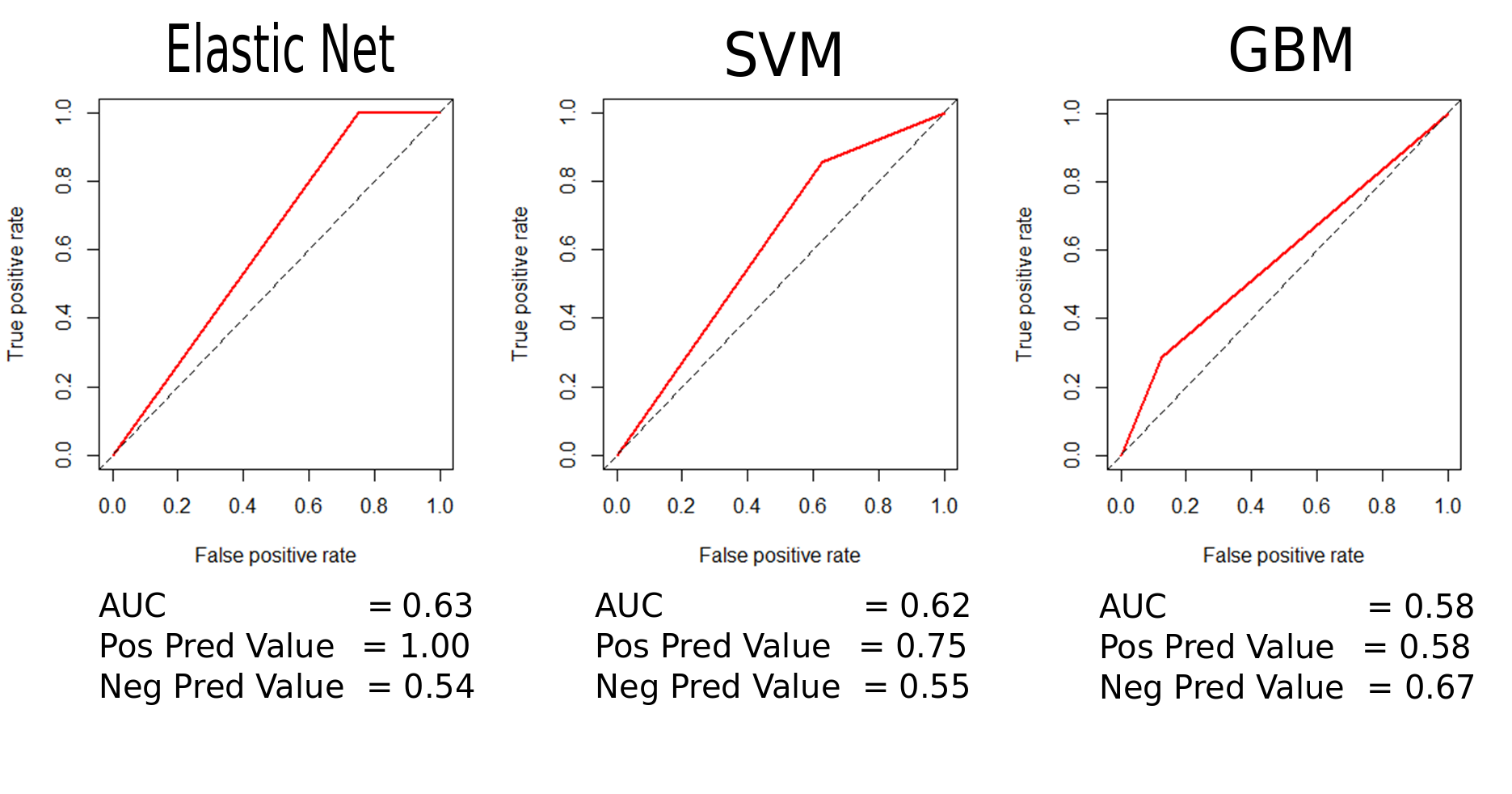

Supplement: Supplementary file 1 [file DataSheet_1.docx]
